# Renal pelvis urobiome dysbiosis is associated with postoperative systemic inflammatory response syndrome after percutaneous nephrolithotomy

**DOI:** 10.1128/msystems.00780-25

**Published:** 2025-08-15

**Authors:** Qing Wang, Xiaolong Chen, Guanyun Deng, Kunyuan Huang, Senyuan Hong, Kehua Jiang

**Affiliations:** 1 Department of Urology, Guizhou Provincial People’s Hospital, Guiyang, Guizhou, China; 2 Department of Urology, Tongji Hospital, Tongji Medical College, Huazhong University of Science and Technology 12443, Wuhan, Hubei, China; Institut de Recherche pour le Developpement Delegation Regionale Occitanie, Montpellier, France

**Keywords:** percutaneous nephrolithotomy, systemic inflammatory response syndrome, urobiome, 2bRAD-M

## Abstract

Early prediction and diagnosis of systemic inflammatory response syndrome (SIRS) following percutaneous nephrolithotomy (PCNL) are critical. This study aimed to investigate differences in clinical characteristics and the renal pelvis urobiome between patients with and without post-PCNL SIRS to identify potential predictive biomarkers. Patients undergoing unilateral PCNL were categorized into SIRS(+) and SIRS(−) groups based on postoperative SIRS status. Renal pelvis urine samples were collected for 2bRAD-M sequencing to profile the urobiome. Clinical data and urobiome composition were compared between the groups. Logistic regression identified preoperative serum albumin-globulin ratio (AGR) as an independent protective factor and operative time as an independent risk factor for post-PCNL SIRS, with an area under the receiver operating characteristic curve (AUC) of 0.76. Diversity analysis revealed distinctive microbial differences between the two groups. Through differential analysis and random forest, we screened six species, including *Sphingomonas paucimobilis*, *Ralstonia *sp000620465, *Ralstonia pickettii*, *Pelomonas puraquae*, *Comamonas tsuruhatensis*, and *Lawsonella clevelandensis_A*, to build the microbial prediction model, which achieved an AUC of 0.81. The combination of microbial data and clinical factors further improved predictive accuracy, achieving an AUC of 0.94. Functional profiling of the urobiome also demonstrated significant intergroup differences. This is the first study to explore renal pelvis urobiome dysbiosis in post-PCNL SIRS. Both clinical and microbial factors showed strong predictive value, with their combination offering the greatest discriminatory power. This research could pave the way for the early prediction of post-PCNL SIRS.

**IMPORTANCE:**

Given the significant morbidity associated with postoperative percutaneous nephrolithotomy (PCNL) systemic inflammatory response syndrome (SIRS), early prediction and diagnosis are crucial for preventing severe complications like sepsis, which can lead to multiple organ dysfunction or death. Our study uniquely explores how renal pelvis urobiome dysbiosis contributes to post-PCNL SIRS. By utilizing the novel 2bRAD-M sequencing, the research identifies key microbial species in the renal pelvis and integrates them with clinical factors like albumin-globulin ratio and operative time. The resulting prediction model, with an impressive area under the curve, significantly outperforms traditional clinical models. This offers a more precise approach to stratify patients at high risk of developing SIRS. This work suggests that microbial imbalances may actively drive SIRS, pointing to the potential to revolutionize the predictive strategies for post-PCNL SIRS.

## INTRODUCTION

Percutaneous nephrolithotomy (PCNL), a minimally invasive surgical technique, is recommended as the first-line treatment for renal stones, especially for large and complex stones, due to excellent stone clearance rates (1, 2). Compared with extracorporeal shock wave lithotripsy and ureteroscopic lithotripsy, PCNL involves greater invasiveness, which is associated with relatively higher postoperative complication rates (3). Systemic inflammatory response syndrome (SIRS) is a common complication after PCNL, with an incidence of up to 35% in patients with complex stones (4). Without timely and effective treatment, SIRS might progress to sepsis, with an incidence of 0.3–7.6%, and severe sepsis can lead to multiple organ dysfunction and death (5, 6).

Numerous studies have been conducted to explore the risk factors and establish the prediction model for post-PCNL SIRS, all of which focus on demographic (gender, age, body mass index [BMI], etc.), preoperative (hematology, blood chemistry, urinalysis, imaging, etc.), and intraoperative data (operative time, channel number, etc.) (7–9). A meta-analysis comprehensively identified several independent risk factors for post-PCNL SIRS, including female sex, elevated peripheral blood leukocytes, multiple puncture channels, and prolonged operation time (10). Notably, positive urine leukocytes, positive urine/renal pelvis urine/stone cultures were also identified as risk factors (10), suggesting that urinary tract infections (UTIs) are associated with an increased risk of post-PCNL SIRS, and urinary microbes might play a crucial role in post-PCNL SIRS.

With breakthroughs in sequencing and culturomics, the existence of a vast and diverse community of microbes has been revealed within the urinary tract (11). The urinary microbiome (urobiome) was found to be associated with kidney stone disease, which showed marked differences between stone formers and healthy individuals (12, 13). On the other hand, specific gut microbiota signatures may allow for predicting the occurrence and progression of sepsis (14, 15). However, these studies utilize data from the gut microbiome and lack research on the urobiome. It is possible to speculate that the risk of post-PCNL SIRS might be partially attributed to microbial dysbiosis in the urobiome.

To address this gap, we conducted an observational cohort study of kidney stone patients admitted to our hospital. Renal pelvis urine samples were collected from patients before PCNL and subjected to 2bRAD-M to characterize the renal pelvis urobiome. 2bRAD-M was selected because it enables high-resolution, species-level profiling in low-biomass microbiome like renal pelvis urine, with minimal DNA input requirements. Besides, demographic, preoperative, and intraoperative clinical data were also collected. This study aimed to explore the differences in clinical characteristics and renal pelvis urobiome between patients with or without SIRS, to identify potentially effective clinical factors and microbial biomarkers for SIRS risk prediction, and to provide evidence-based insights for early prevention and treatment of SIRS.

## MATERIALS AND METHODS

### Study design and participants

The study was approved by the Ethics Committee of Guizhou Provincial People’s Hospital (ethical number: [2021]42) and conducted from July to December 2022. Kidney stone patients were recruited when they were diagnosed by computed tomography and received unilateral PCNL, and patients were excluded when (i) they would receive bilateral PCNL; (ii) had previous a nephrostomy tube on the operative side; (iii) had urinary malformation; and (iv) had urinary tuberculosis. A total of 123 kidney stone patients were asked for permission to obtain their renal pelvis urine samples before PCNL. Patients were excluded from further analysis for the following situations: (i) insufficient urine; (ii) polluted urine (when the urine sample came into contact with a non-sterile environment during collection); (iii) incomplete clinical data; (iv) nephrostomy only; and (v) tubeless PCNL. Ultimately, a total of 96 patients were enrolled in the study, and their renal pelvis urine samples were sequenced by 2bRAD-M. Patients’ demographic data, including age, gender, BMI, hypertension, and diabetes; preoperative data, including urinary leukocyte, urinary nitrite, peripheral leukocyte, peripheral neutrophil-to-lymphocyte ratio (NLR), peripheral platelet-to-lymphocyte ratio (PLR), peripheral lymphocyte-to-monocyte ratio (LMR), blood urea nitrogen (BUN), serum creatinine, estimated glomerular filtration rate (eGFR), albumin-to-globulin ratio (AGR), stone volume, stone mean Hounsfield unit (HU), and operative side; and intraoperative data, including ASA score, channel size, and operative time were collected. Preoperative urine cultures were obtained as a routine procedure, and all patients showed negative results for these cultures. In order to avoid the influence of antibiotics on the urine microbiome, no antibiotics were administered to patients before PCNL.

**Fig 1:**
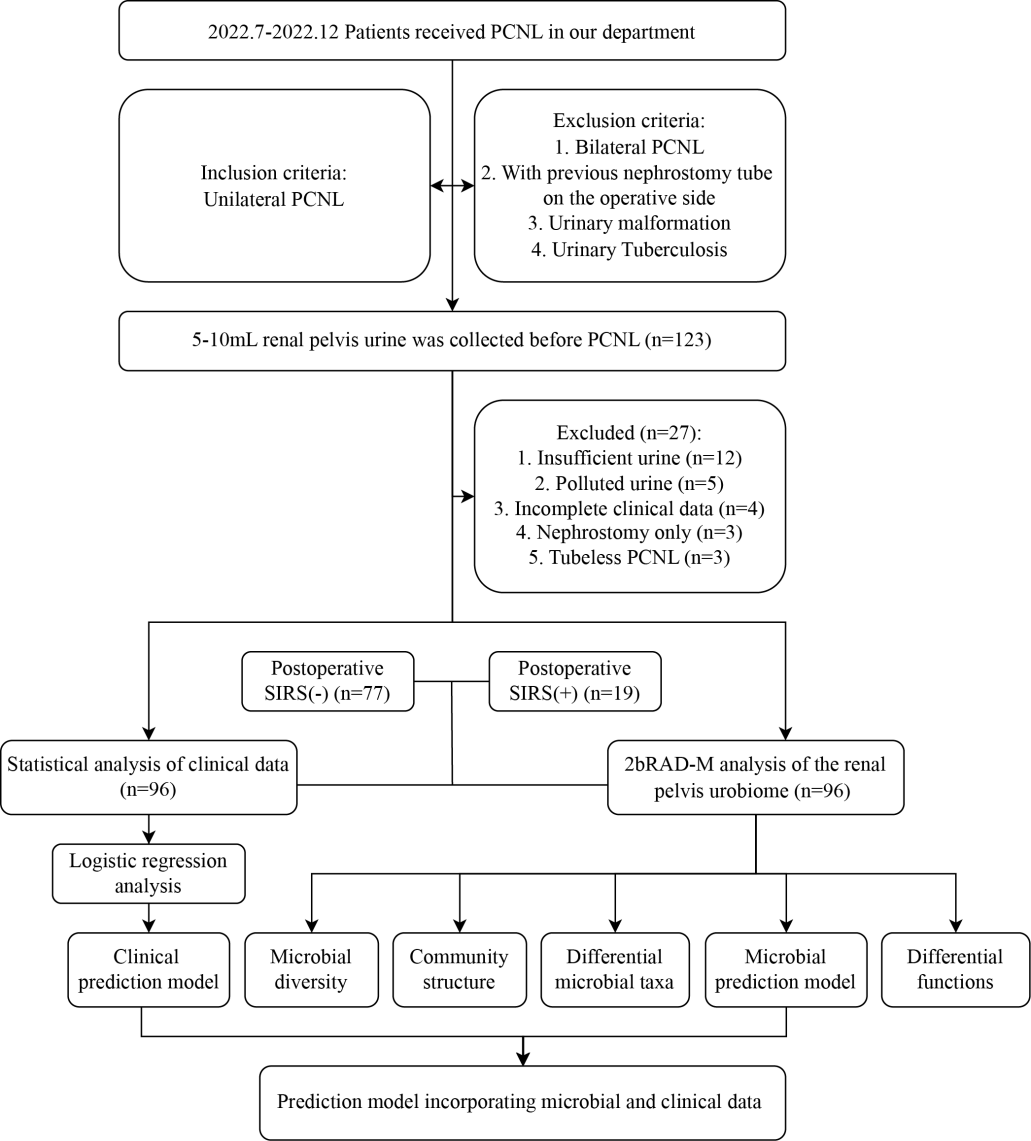
The flowchart of the study design.

All patients were followed up postoperatively for SIRS criteria. SIRS was defined as meeting any two or more of the following four criteria: (i) body temperature of >38°C or <36°C; (ii) heart rate of >90 beats/min; (iii) respiratory rate of >20 breaths/min or partial pressure of carbon dioxide in arterial blood of <32 mmHg; (iv) leucocyte count of <4,000 or >12,000 cells/µL (16). Patients who developed SIRS were categorized into the SIRS(+) group, while patients who did not develop SIRS were categorized into the SIRS(−) group. The flowchart of the study design is illustrated in Fig. 1.

### Sample collection and processing

All PCNL cases were performed by the same surgical team and lead surgeon. Following the induction of general anesthesia, an open-end 6F ureteral catheter was placed transurethrally using a cystoscope in the lithotomy position. The patient was then repositioned to the prone position. Percutaneous access was achieved via puncturing with an 18-gauge needle under ultrasound guidance. Upon successful puncture into the pelvicalyceal system, 5–10 mL renal pelvis urine was collected before expansion and lithotripsy. The urine samples were stored in −80°C within 1 h from collection. The whole process was performed under aseptic conditions.

### 2bRAD-M sequencing and quantitative analysis

The genomic DNA from renal pelvis urine samples was extracted using a TIANamp Micro DNA Kit (Tiangen, China) and used for 2bRAD-M library construction according to the previously described protocol (17). The library products were purified and then subjected to sequencing using the Illumina Nova PE150 platform. To identify microbial species within each sample, all sequenced 2bRAD tags were subjected to quality control and subsequently aligned against the 2bRAD marker database, which encompasses all 2bRAD tags theoretically unique to each of 86,022 microbial species in the database. To control the false positives in species identification, the *G* score was derived for each species identified within a sample using the following formula: *G* score _species *i*_ = $\sqrt{S_{i}\times t_{i}}$ (*S*: the number of reads assigned to all 2bRAD markers belonging to species *i* within a sample; *t*: the number of all 2bRAD markers of species *i* that have been sequenced within a sample). A threshold *G* score of 5 was set for a false-positive discovery of microbial species. Then, the relative abundance of a given species was calculated with the following formula: Relative abundance _species *i*_ = $\frac{S_{i}/T_{i}}{\sum_{i=1}^{n}S_{i}/T_{i}}$ (*S*: the number of reads assigned to all 2bRAD markers of species *i* within a sample; *T*: the number of all theoretical 2bRAD markers of species *i*). Finally, the taxonomic relative abundance profile was generated. 2bRAD-M was carried out at the Qingdao OE Biotech Co., Ltd. (Qingdao, China).

Of note, we included a total of four negative controls at different stages: two collected during sampling (surgical instrument swabs) and two during laboratory procedures (blank controls for DNA extraction and library preparation reagents and consumables). These served to detect and exclude potential environmental or reagent-derived contaminants. Sequencing data showed that clinical samples yielded an average of 7.4 million reads per sample, while the negative controls during sampling and laboratory procedures yielded averages of only 2,400 and 7,500 reads per sample, respectively. The results indicate minimal contamination during these stages. Furthermore, microbial profiling identified two bacterial species in the negative controls: *Streptococcus mutans* and *Streptococcus agalactiae*. Neither species was detected in any clinical sample. Therefore, the contamination present during collection and laboratory processing had no significant effect on the results.

### Bioinformatics and statistical analysis

For clinical data, normally distributed continuous variables were expressed as mean ± standard deviation and analyzed by Student’s *t*-test, and non-normally distributed continuous variables were expressed as median (interquartile range) and analyzed by Wilcoxon rank-sum test. Categorical variables were reported as percentages (%) and analyzed by the chi-square test or Fisher’s exact test. Univariate and multivariate logistic regression analyses were used for risk factor analysis. Variables with a *P* value < 0.05 in the univariate analysis were included in multivariate logistic regression analysis to determine the independent risk factors for post-PCNL SIRS. The receiver operating characteristic (ROC) curve was constructed, and the area under the ROC curve (AUC) was calculated to evaluate the discriminatory ability of the logistic regression model.

For microbial diversity analysis, alpha diversity indices including Chao1, Shannon, and Simpson index were calculated using the “vegan” package and visualized as boxplots; beta diversity was estimated by computing Bray-Curtis, binary-Jaccard, and Euclidean distance algorithms using the “vegan” package and visualized as principal coordinate analysis (18). Group comparisons of alpha diversity were analyzed by the Wilcoxon rank-sum test, and group comparisons of beta diversity were analyzed by permutational multivariate analysis of variance.

For microbial differential analysis, we first used the Wilcoxon rank-sum test to compare the relative abundance of different taxa between the two groups. Multiple approaches were also applied to identify differential microbial genera and species. Linear discriminant analysis (LDA) of effect size (LEfSe) determines the features most likely to explain differences between groups by combining the standard tests for statistical significance (Kruskal-Wallis test or Wilcoxon rank-sum test) with LDA for encoding biological consistency and effect relevance (19). MaAsLin2 (Microbiome Multivariable Associations with Linear Models 2) relies on general linear models to efficiently determine multivariable associations between clinical data and microbial features and identify disease-associated features (20). Indicator species analysis is a method for calculating the indicator values of each species to screen the indicator species whose status provides information on the overall condition of the ecosystem and reflects the community composition (21).

Random forest is a machine learning algorithm that can effectively and accurately classify microbial community samples and identify key species that can distinguish intergroup differences (22). The ROC curve and AUC were generated to evaluate the ability of the microbial prediction model. In addition, the correlation between differential species and preoperative clinical data were calculated using the Spearman correlation analysis. The prediction model incorporating microbial and clinical data was also evaluated by ROC curve and AUC. Finally, PICRUSt2 was used to predict functional profiles of the microbial communities, and the functional differences between groups were analyzed by the Wilcoxon rank-sum test (23). It is worth noting that PICRUSt2 predictions are inferential and based on marker gene fragments, which may introduce limitations in accuracy. Statistical and bioinformatic analysis was performed using SPSS (version 26) and R software (version 4.1.1). A *P* value < 0.05 was considered statistically significant.

## RESULTS

### Clinical outcomes of the enrolled patients

**Fig 2:**
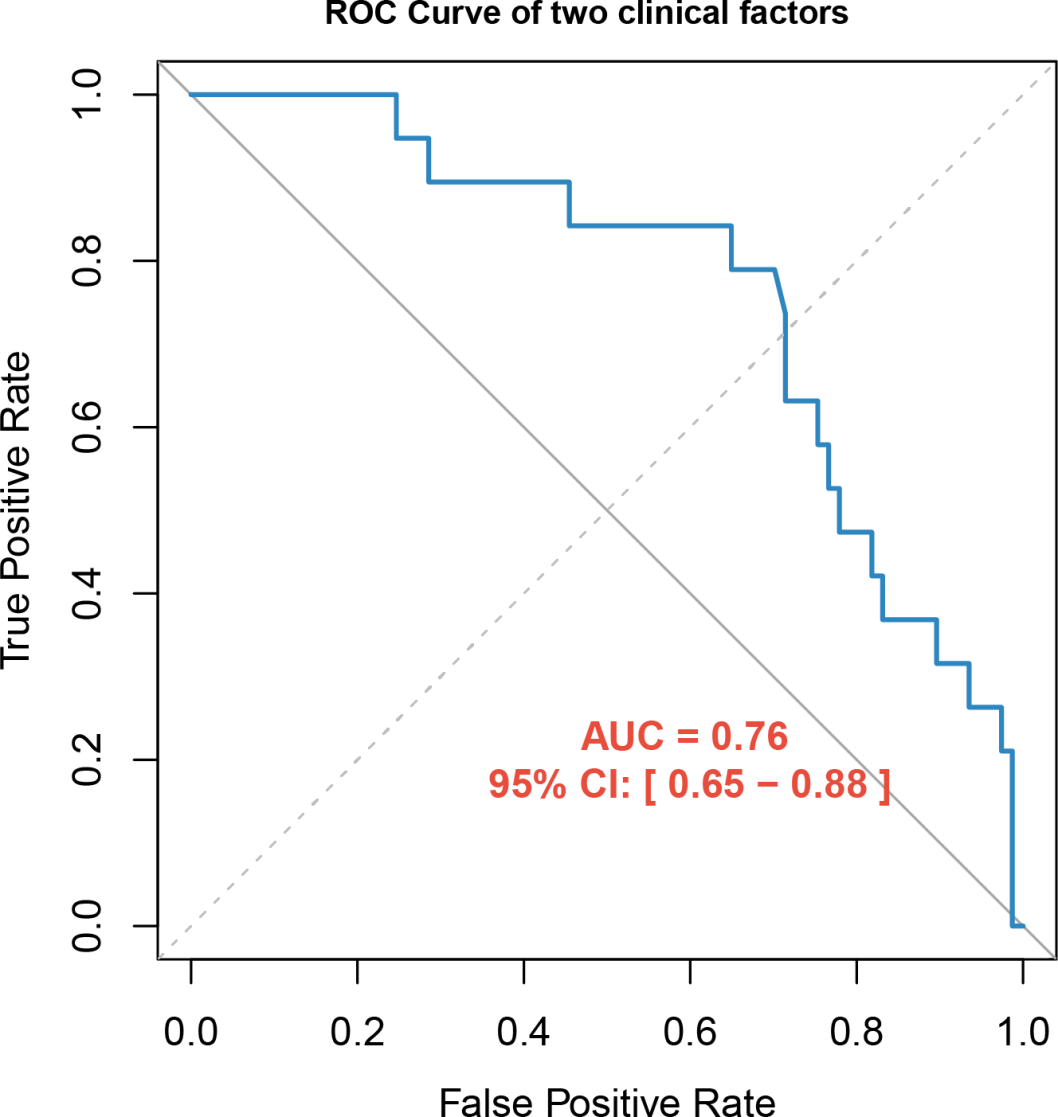
The ROC curve of the clinical prediction model combining AGR and operative time.

A total of 96 kidney stone patients (57 males and 39 females) were included in the study, with the median age being 48.5 years. Nineteen cases developed SIRS after PCNL, accounting for 19.8% of the total. The detailed demographic and clinical characteristics of the enrolled patients are listed in Table 1. No statistically significant difference was found between the two groups in age, BMI, gender, hypertension, diabetes, urinary nitrite, peripheral leukocyte, NLR, PLR, LMR, BUN, creatinine, eGFR, ASA score, stone volume, stone mean HU, operative side, and channel size. Compared with the SIRS(−) group, the SIRS(+) group had a higher preoperative urinary leukocyte count, a lower preoperative AGR, and longer operative time. The results of univariate logistic regression analysis showed that the factors significantly correlated with post-PCNL SIRS included AGR (odds ratio [OR] = 0.074, 95% confidence interval [CI] = [0.007–0.742], *P* = 0.027) and operative time (OR = 1.025, 95% CI = [1.007–1.042], *P* = 0.005) (Table 2). The two factors were subjected to multivariate logistic regression analysis. The results showed that AGR (OR = 0.084, 95% CI = [0.008–0.898], *P* = 0.040) and operative time (OR = 1.024, 95% CI = [1.007–1.042], *P* = 0.007) were independent protective and risk factors for post-PCNL SIRS (Table 2). The ROC curve of the clinical prediction model combining AGR and operative time showed an AUC of 0.76 (Fig. 2).

**TABLE 1:** Demographic and clinical characteristics of the enrolled patients (*N* = 96)^*a*^

Parameter	Overall (*N* = 96)	SIRS(−) (*N* = 77)	SIRS(+) (*N* = 19)	*P* value
Age (years), median (IQR)	48.50 (39.75–59.00)	47.00 (40.00–59.00)	52.00 (40.50–59.00)	0.559
BMI (kg/m^2^), median (IQR)	23.57 (21.35–27.59)	23.67 (21.80–27.64)	23.44 (19.26–26.91)	0.398
Gender, *N* (%)				0.353
Male	57 (59.38%)	48 (62.34%)	9 (47.37%)	
Female	39 (40.63%)	29 (37.66%)	10 (52.63%)	
Hypertension, *N* (%)				0.435
No	70 (72.92%)	58 (75.32%)	12 (63.16%)	
Yes	26 (27.08%)	19 (24.68%)	7 (36.84%)	
Diabetes, *N* (%)				0.451
No	85 (88.54%)	69 (89.61%)	16 (84.21%)	
Yes	11 (11.46%)	8 (10.39%)	3 (15.79%)	
Urinary leukocyte (/μL), median (IQR)	84.00 (19.50–237.75)	63.00 (15.00–159.00)	219.00 (112.00–971.50)	0.002
Urinary nitrite, *N* (%)				0.105
Negative	86 (89.58%)	71 (92.21%)	15 (78.95%)	
Positive	10 (10.42%)	6 (7.79%)	4 (21.05%)	
Peripheral leukocyte (10^9^/L), mean ± SD	7.21 ± 1.99	7.20 ± 1.94	7.27 ± 2.23	0.900
Peripheral NLR, median (IQR)	2.21 (1.65–3.02)	2.13 (1.63–2.93)	2.28 (1.86–3.24)	0.652
Peripheral PLR, median (IQR)	117.95 (94.19–156.80)	117.36 (93.26–167.91)	128.53 (97.51–151.18)	0.613
Peripheral LMR, median (IQR)	4.11 (3.21–4.94)	4.09 (3.21–4.93)	4.21 (3.17–4.91)	0.949
BUN (mmol/L), median (IQR)	5.28 (4.15–6.43)	5.26 (4.04, 6.36)	5.30 (4.39, 6.54)	0.454
Creatinine (μmol/L), median (IQR)	85.00 (69.75–101.75)	85.00 (71.00–101.00)	76.00 (64.00–130.00)	0.734
eGFR (mL/min/1.73 m^2^), mean ± SD	81.79 ± 27.12	82.33 ± 24.85	79.58 ± 35.63	0.753
AGR, mean ± SD	1.51 ± 0.23	1.53 ± 0.21	1.40 ± 0.28	0.036
ASA score, *N* (%)				0.700
II	84 (87.50%)	68 (88.31%)	16 (84.21%)	
III	12 (12.50%)	9 (11.69%)	3 (15.79%)	
Stone volume (cm^3^), median (IQR)	1.66 (0.83–3.98)	1.30 (0.83–3.95)	2.85 (1.09–4.29)	0.353
Stone mean HU, mean ± SD	676.78 ± 215.83	688.83 ± 196.07	627.95 ± 283.51	0.385
Operative side, *N* (%)				
Left	53 (55.21%)	42 (54.55%)	11 (57.89%)	0.996
Right	43 (44.79%)	35 (45.45%)	8 (42.11%)	
Channel size, *N* (%)				0.756
16F	66 (68.75%)	54 (70.13%)	12 (63.16%)	
18F	30 (31.25%)	23 (29.87%)	7 (36.84%)	
Operative time (min), median (IQR)	75.00 (59.75–102.25)	70.00 (55.00–90.00)	100.00 (79.50–119.00)	0.004

^*a*^ AGR, albumin-to-globulin ratio; ASA, American Society of Anesthesiologists; BMI, body mass index; BUN, blood urea nitrogen; eGFR, estimated glomerular filtration rate; HU, Hounsfield unit; IQR, interquartile range; LMR, lymphocyte-to-monocyte ratio; NLR, neutrophil-to-lymphocyte ratio; PLR, platelet-to-lymphocyte ratio; SD, standard deviation.

**TABLE 2:** Univariate and multivariate analyses of clinical data for predicting SIRS after PCNL^*a*^

Variable	Univariate analysis			Multivariate analysis		
	OR	95% CI	*P* value	OR	95% CI	*P* value
Age (years)	1.012	0.973–1.052	0.551	–^*b*^	–	–
BMI (kg/m^2^)	0.947	0.830–1.081	0.422	–	–	–
Gender, female	1.839	0.669–5.058	0.238	–	–	–
Hypertension, yes	1.781	0.613–5.173	0.289	–	–	–
Diabetes, yes	1.617	0.385–6.785	0.511	–	–	–
Urinary leukocyte (/μL)	1.000	1.000–1.001	0.061	–	–	–
Urinary nitrite, positive	3.156	0.792–12.572	0.103	–	–	–
Peripheral leukocyte (10^9^/L)	1.018	0.791–1.311	0.888	–	–	–
Peripheral NLR	1.046	0.796–1.374	0.747	–	–	–
Peripheral PLR	1.000	0.992–1.007	0.961	–	–	–
Peripheral LMR	0.944	0.742–1.201	0.640	–	–	–
BUN (mmol/L)	1.150	0.940–1.405	0.174	–	–	–
Creatinine (μmol/L)	1.009	0.997–1.021	0.136	–	–	–
eGFR (mL/min/1.73 m^2^)	0.996	0.978–1.015	0.690	–	–	–
AGR	0.074	0.007–0.742	0.027	0.084	0.008–0.898	0.040
ASA score, III	1.417	0.344–5.835	0.630	–	–	–
Stone volume (cm^3^)	0.999	0.902–1.106	0.987	–	–	–
Stone mean HU	0.999	0.996–1.001	0.271	–	–	–
Stone side, left	1.146	0.415–3.162	0.793	–	–	–
Channel size, 18F	1.370	0.478–3.922	0.558	–	–	–
Operative time (min)	1.025	1.007–1.042	0.005	1.024	1.007–1.042	0.007

^*a*^ AGR, albumin-to-globulin ratio; ASA, American Society of Anesthesiologists; BMI, body mass index; BUN, blood urea nitrogen; CI, confidence interval; eGFR, estimated glomerular filtration rate; HU, Hounsfield unit; LMR, lymphocyte-to-monocyte ratio; NLR, neutrophil-to-lymphocyte ratio; PLR, platelet-to-lymphocyte ratio.

^*b*^ "–” indicates not applicable.

### Diversity of the renal pelvis urobiome between post-PCNL SIRS(−) and SIRS(+)

**Fig 3:**
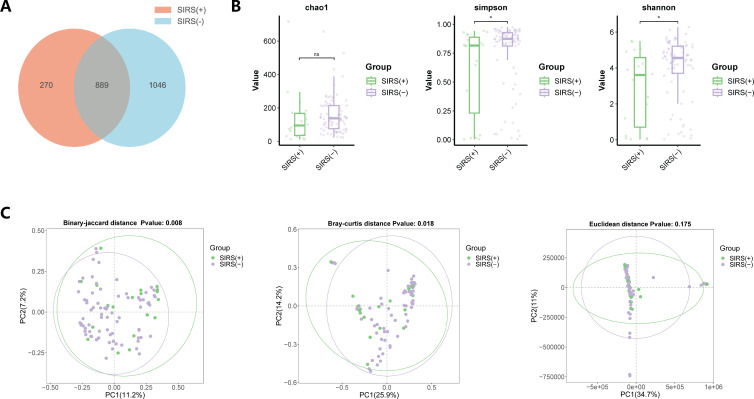
Microbial diversity of the renal pelvis urobiome between the SIRS(+) group and the SIRS(−) group. (**A**) The Venn diagram exhibited the shared and unique species between the two groups. (**B**) Comparison of alpha diversity (Chao1, Simpson, and Shannon index) between the two groups. (**C**) Comparison of beta diversity between the two groups based on binary-Jaccard distance, Bray-Curtis distance, and Euclidean distance.

A total of 2,205 species were identified from 96 renal pelvis urine samples. The SIRS(+) group and the SIRS(−) group shared 889 (40.32%) species in common. Two hundred seventy (12.24%) unique species were identified in the SIRS(+) group, and 1,046 (47.44%) in the SIRS(−) group (Fig. 3A). For alpha diversity, the Chao1 index was calculated to evaluate community richness, and the Shannon index and Simpson index were calculated to assess community diversity. The Chao1 index (0.077) showed no significant difference between the two groups, while the Shannon index (0.014) and Simpson index (0.015) were higher in the SIRS(−) group (Fig. 3B). For beta diversity, significant differences were found in the Binary-Jaccard distance (*P* = 0.008) and Bray-Curtis distance (*P* = 0.018) between the two groups, but no significant differences were observed in the Euclidean distance (*P* = 0.175) (Fig. 3C).

### Community structure of the renal pelvis urobiome between post-PCNL SIRS(−) and SIRS(+)

**Fig 4:**
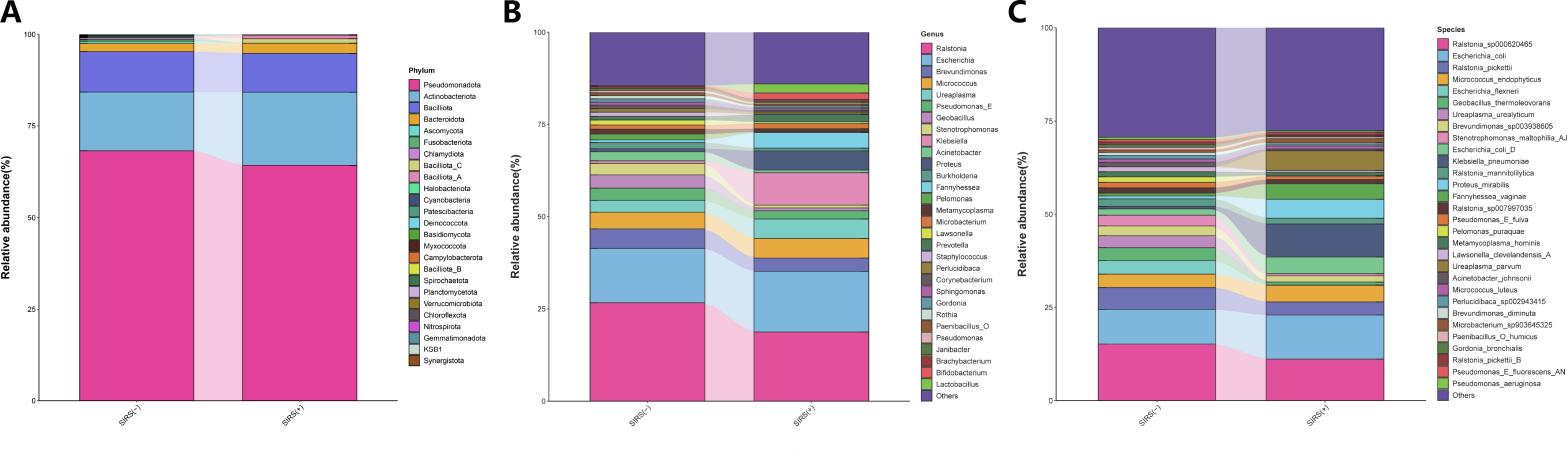
Community structure of the renal pelvis urobiome between the SIRS(+) group and the SIRS(−) group. The relative abundance of the microbial phyla (**A**), the top 30 most abundant genera (**B**), and species (**C**) were represented in the barplot.

Twenty-one and 22 phyla were identified in the SIRS(+) and SIRS(−) groups, respectively. Pseudomonadota (64.26 and 68.24%), Actinobacteriota (19.94 and 16.01%), and Bacillota (10.61 and 11.03%) were the top three most abundant phyla for both groups (Fig. 4A). Three hundred thirty-one and 527 genera were identified in the SIRS(+) and SIRS(−) groups, respectively. The top five most abundant genera in the SIRS(+) group were *Ralstonia* (18.72%), *Escherichia* (16.40%), *Klebsiella* (8.83%), *Micrococcus* (5.32%), and *Ureaplasma* (5.28%), and the top five most abundant genera in the SIRS(−) group were *Ralstonia* (26.63%), *Escherichia* (14.72%), *Brevundimonas* (5.28%), *Micrococcus* (4.51%), and *Geobacillus* (3.59%) (Fig. 4B). At the species level, *Escherichia coli* (11.81%), *Ralstonia sp000620465* (11.12%), *Klebsiella pneumoniae* (8.83%), *Ureaplasma parvum* (5.28%), and *Proteus mirabilis* (5.16%) were the top five most abundant species in the SIRS(+) group, while *Ralstonia *sp000620465 (15.16%), *E. coli* (9.27%), *Ralstonia pickettii* (5.89%), *Escherichia flexneri* (3.68%), and *Micrococcus endophyticus* (3.56%) were the top five most abundant species in the SIRS(−) group (Fig. 4C).

### Differential microbial taxa between post-PCNL SIRS(−) and SIRS(+)

**Fig 5:**
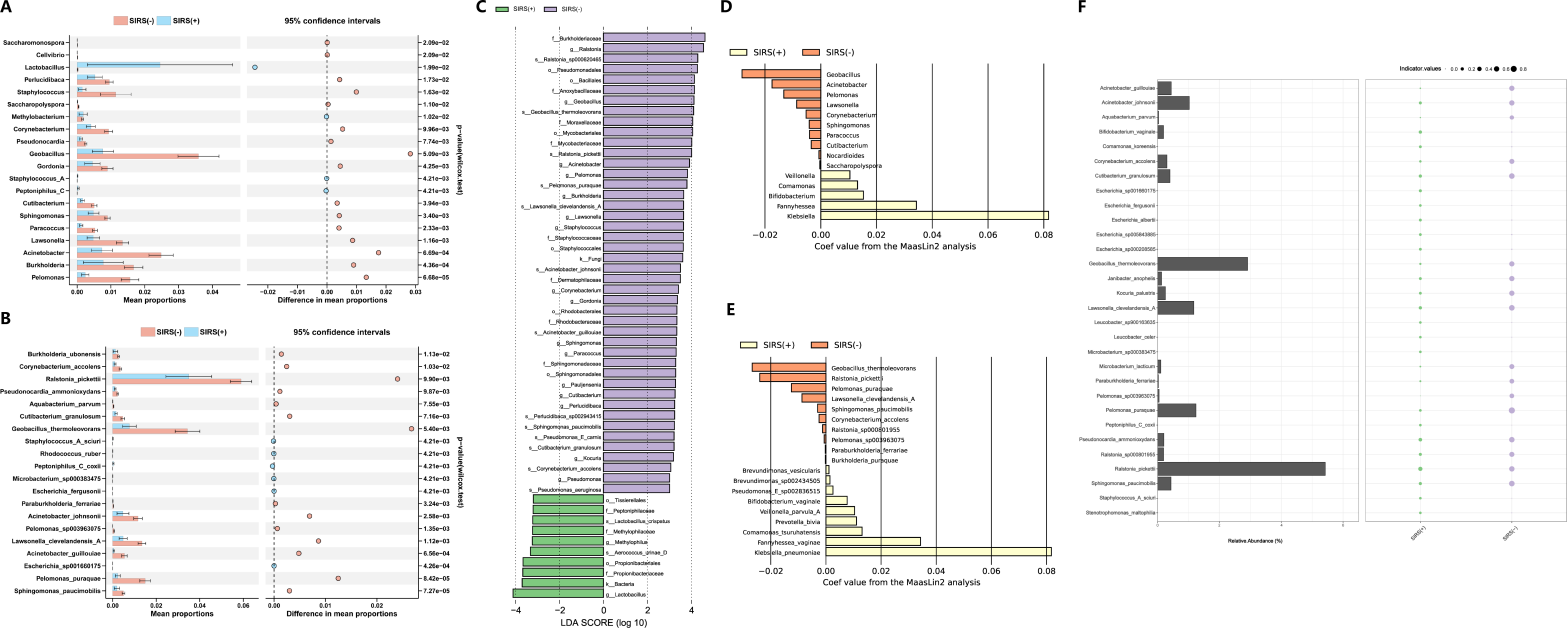
Differential microbial taxa between the SIRS(+) group and the SIRS(−) group. (**A**) Wilcoxon rank-sum test comparison demonstrated the top 20 differential genera. (**B**) Wilcoxon rank-sum test comparison demonstrated the top 20 differential species. (**C**) LEfSe identified 54 discriminative taxa with significant differences between the two groups. LDA score represented the influencing degree of microbial taxa. (**D**) Fifteen differential genera were calculated with MaAsLin2 and displayed in the barplot. (**E**) Nineteen differential species were calculated with MaAsLin2 and displayed in the barplot. (**F**) Indicator species analysis identified 30 indicator species, and their indicator values were presented.

First, 97 differential genera and 320 differential species between the two groups were identified by the Wilcoxon rank-sum test (Table S1). Top 20 genera with the most significant *P* value are presented in Fig. 5A. For example, the SIRS(−) group exhibited increased abundance of *Pelomonas*, *Burkholderia*, and *Acinetobacter* and decreased abundance of *Peptoniphilus_C*, *Staphylococcus_A*, and *Methylobacterium*. Top 20 species with the most significant *P* value are presented in Fig. 5B. For example, the abundance of *Sphingomonas paucimobilis*, *Pelomonas puraquae*, *Acinetobacter guillouiae*, *Lawsonella clevelandensis_A*, *Pelomonas *sp003963075, and *Acinetobacter johnsonii* was higher in the SIRS(−) group, while the abundance of *Escherichia *sp001660175, *Escherichia fergusonii*, *Microbacterium *sp000383475, *Peptoniphilus_C coxii*, *Rhodococcus ruber*, and *Staphylococcus_A sciuri* was higher in the SIRS(+) group.

Next, LEfSe identified 54 discriminative features (LDA score ≥ 3.0) between the two groups, including 15 species (Fig. 5C). The SIRS(−) group was enriched with *Ralstonia *sp000620465, *Geobacillus thermoleovorans*, *R. pickettii*, *P. puraquae*, *L. clevelandensis_A*, *A. johnsonii*, *A. guillouiae*, *Perlucidibaca *sp002943415, *S. paucimobilis*, *Pseudomonas E_carnis*, *Cutibacterium granulosum*, *Corynebacterium accolens*, and *Pseudomonas aeruginosa*, while the SIRS(+) group was enriched with *Aerococcus urinae_D* and *Lactobacillus crispatus*.

Meanwhile, MaAsLin2 identified 15 differential genera and 19 differential species between the two groups. The top five enriched genera in the SIRS(−) group were *Geobacillus*, *Acinetobacter*, *Pelomonas*, *Lawsonella*, and *Corynebacterium*, while the top five enriched genera in the SIRS(+) group were *Klebsiella*, *Fannyhessea*, *Bifidobacterium*, *Comamonas*, and *Veillonella* (Fig. 5D). The top five enriched species in the SIRS(−) group were *G. thermoleovorans*, *R. pickettii*, *P. puraquae*, *L. clevelandensis_A*, and *S. paucimobilis*, while the top five enriched species in the SIRS(+) group were *K. pneumoniae*, *Fannyhessea vaginae*, *Comamonas tsuruhatensis*, *Prevotella bivia*, and *Veillonella parvula_A* (Fig. 5E).

Besides, indicator species analysis identified a total of 30 indicator species, including 13 species dominant in the SIRS(+) group and 17 species dominant in the SIRS(−) group (Fig. 5F). The top five indicator species in the SIRS(+) group were *Escherichia *sp001660175, *Escherichia albertii*, *Bifidobacterium vaginale*, *S._A sciuri*, and *E. fergusonii*. The top five indicator species in the SIRS(-) group were *P. puraquae*, *S. paucimobilis*, *L. clevelandensis_A*, *Pelomonas *sp003963075, and *G. thermoleovorans*.

### Classification of post-PCNL SIRS status based on microbial biomarkers

**Fig 6:**
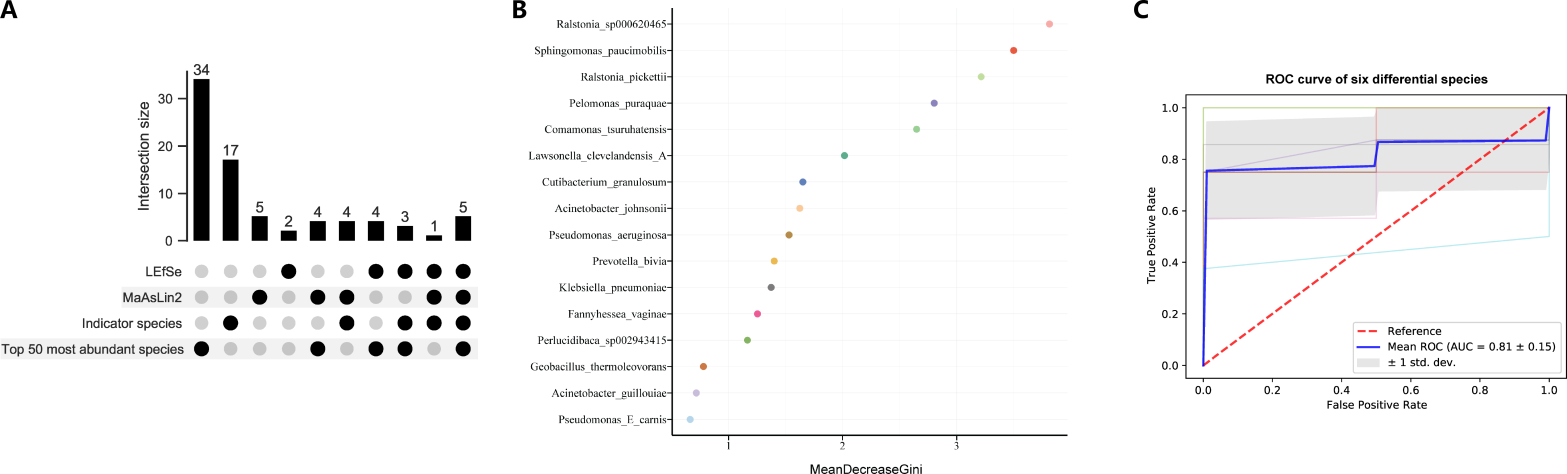
Classification of post-PCNL SIRS status based on microbial biomarkers. (**A**) The Upset diagram was applied to screen microbial biomarkers. (**B**) Random forest analysis showed the MDG of 16 species. (**C**) The ROC curve of the microbial prediction model included six species.

The top 50 most abundant species that were identified as differential species by at least one approach were selected for random forest analysis, and a total of 16 species were included (Fig. 6A). The importance of 16 species was measured by Mean Decrease Gini (MDG), and the species with an MDG greater than two were selected for random forest construction (Fig. 6B). Six species were selected as the optimal marker set to distinguish the two groups, including *Ralstonia *sp000620465, *S. paucimobilis*, *R. pickettii*, *P. puraquae*, *C. tsuruhatensis*, and *L. clevelandensis_A*. The average AUC value achieved 0.81 based on 10-fold cross-validation, suggesting that the microbial prediction model achieved a powerful predictive potential for post-PCNL (Fig. 6C).

### Prediction model incorporating microbial and clinical data

**Fig 7:**
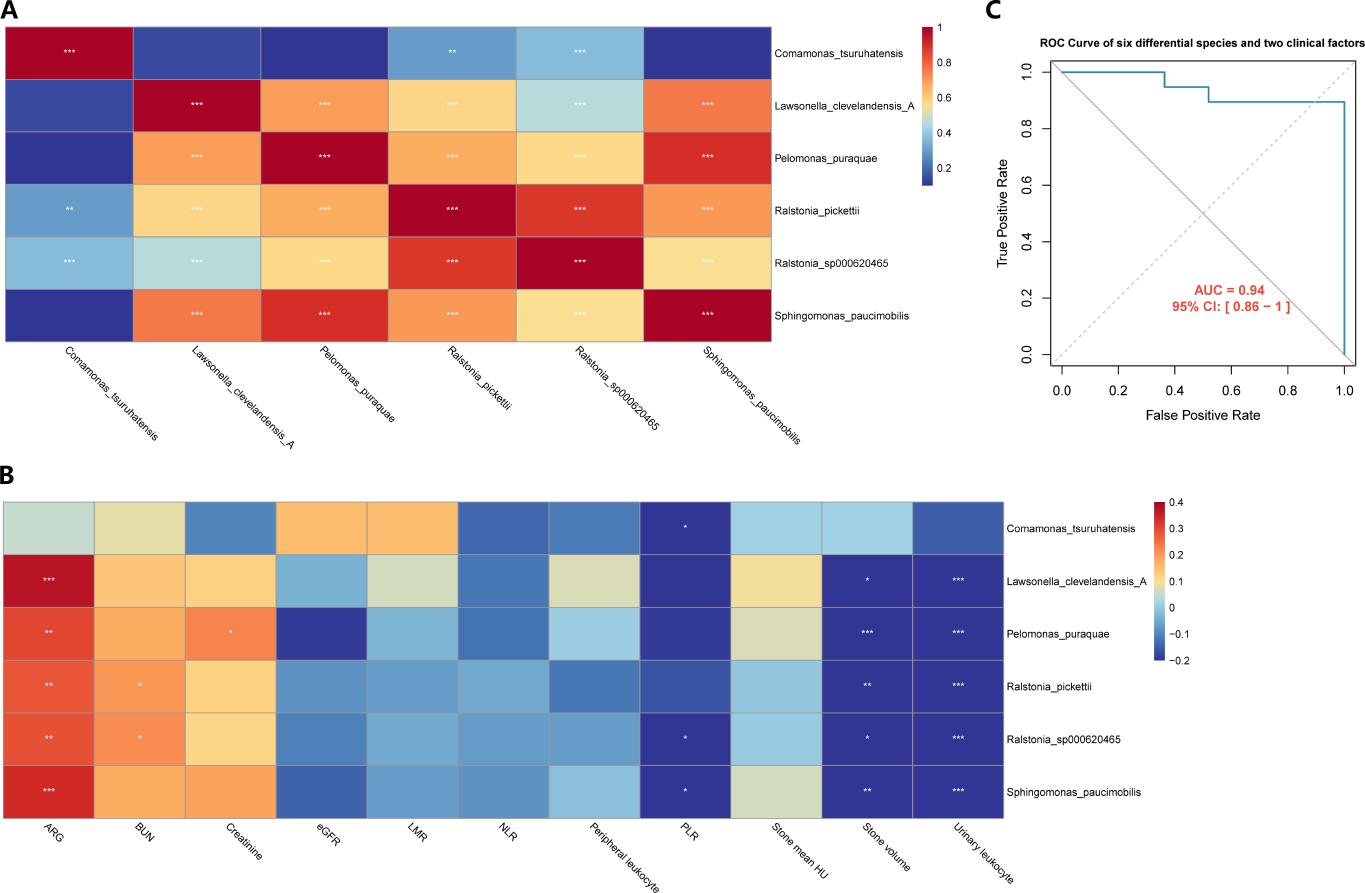
The prediction model incorporating microbial and clinical data. (**A**) Heatmap of Spearman correlation analysis of six differential species. (**B**) Heatmap of Spearman correlation analysis of six differential species and preoperative data. (**C**) The ROC curve of the prediction model combining six differential species and two clinical factors.

Evaluation of the connections among the above selected species was performed by the Spearman correlation analysis. Significant positive correlations were found among these species (Fig. 7A). We further explored the association of the species with preoperative data (Fig. 7B) and found that there were significantly positive correlations between AGR and these species, except for *C. tsuruhatensis*. Of note, stone volume and urinary leukocytes were negatively associated with these species. We constructed the prediction model by combining six differential species, AGR, and operative time, which achieved an ROC of 0.94 (Fig. 7C). To this end, we conclude that microbial biomarkers in combination with clinical protective and risk factors predict post-PCNL SIRS better than any of the two alone.

### Differential predictive functions between post-PCNL SIRS(−) and SIRS(+)

We used PICRUSt2 to predict the functions of the renal pelvis urobiome and found 64 KEGG pathways were differentially enriched between the two groups (Table S2). Sixty pathways were increased in the SIRS(−) group, including calcium signaling pathway, retrograde endocannabinoid signaling, isoflavonoid biosynthesis, staurosporine biosynthesis, and systemic lupus erythematosus. Four pathways were increased in the SIRS(+) group, including bile secretion, photosynthesis-antenna proteins, Jak-STAT signaling pathway, and prolactin signaling pathway.

## DISCUSSION

Our study is the first to characterize the clinical profiles and renal pelvic urobiome in kidney stone patients with or without post-PCNL SIRS. Consistent with previous studies (10, 24), we found that the SIRS(+) group had significantly a lower AGR and a longer operative time than the SIRS(−) group. Logistic regression analysis identified AGR as an independent protective factor and operative time as an independent risk factor for post-PCNL SIRS. On the one hand, preoperative AGR reflects the status of nutrition, systemic inflammation, and immune response. A low AGR indicates nutritional deficiency and leads to the insufficient synthesis of immunoglobulin, which compromises the immune system and increases the risk of postoperative SIRS (25, 26). On the other hand, prolonged operative time is associated with sustained renal pelvic high pressure, venous backflow, and increased absorption of irrigant fluids, all of which heighten the probability of postoperative SIRS (8, 27). Therefore, AGR and operative time can serve as predictors of post-PCNL SIRS. Although the ROC curve of the clinical prediction model combining the two clinical factors achieved an AUC of 0.76, we suspected that some other factors might have better predictive power.

A meta-analysis revealed that patients with positive urine leukocytes, urine/renal pelvis urine/stone cultures were prone to develop post-PCNL SIRS (10), suggesting a potential involvement of urinary microbes and dysbiosis in its pathogenesis. Thus, we assumed that the urobiome might be a reliable predictor. However, existing research predominantly focuses on the relationship between the gut microbiome and SIRS, typically offering taxonomic information only up to the genus level. For example, Du et al. observed higher abundance of *Acinetobacter* and *Enterococcus* and lower abundance of *Roseburia*, *Bacteroides*, *Clostridia*, *Faecalibacterium*, and *Blautia* in septic children compared to healthy controls (28). Liu et al. identified two enterotypes in ICU patients: enterotype I, dominated by *Bacteroides* and an unclassified *Enterobacteriaceae* genus, and enterotype II, characterized by *Enterococcus* predominance (15). They found that different enterotypes were associated with different clinical outcomes, with enterotype I linked to a higher risk of septic shock. Thus, the microbiome could be an effective hallmark for the diagnosis and prognosis. Our study innovatively collected preoperative renal pelvis urine samples from patients and utilized 2bRAD-M to profile the urobiome up to the species level.

Alpha diversity based on the Chao1 index between the two groups approached statistical significance (*P* = 0.077), indicating a tendency toward decreased microbial richness in the SIRS(+) group. In line with prior research (28, 29), alpha diversity based on Simpson and Shannon indices was significantly lower in the SIRS(+) group compared with the SIRS(−) group. The findings implied that a reduction in microbial diversity was linked to an increased risk for SIRS. Beta diversity further showed that the overall microbial composition significantly differed between the two groups. Then, we applied multiple methods to identify differential taxa.

Several species exhibited higher abundance in the SIRS(+) group, indicating a potential causative association with SIRS development. *A. urinae* is a species typically found in the urinary tract, which can cause UTIs and invasive infections such as sepsis and infective endocarditis (30). *E. fergusonii* was reported to cause cognitive impairment through the translocation of byproducts such as lipopolysaccharide (LPS) and peptidoglycan into the brain via the gut-blood-brain axis (31). *E. albertii*, an emerging enteropathogen, can enhance the pathogenicity of *E. coli* by promoting their expression of some virulence factors (32). *S. sciuri* was known for its potent ability to stimulate Nod2, a pattern recognition receptor that detects bacterial components and initiates immune responses (33). *Brevundimonas vesicularis* promotes dendritic cell secretion of IL-6 and TGF-β, driving the differentiation of Treg cells toward Th17 cells and exacerbating skin inflammation (34). Similarly, *V. parvula* was able to induce lower airway inflammation and accelerate lung cancer progression through the recruitment of Th17 cells and neutrophils (35). LPS from *V. parvula* can impair the barrier function of colonic epithelial cells and then activate macrophages to induce intestinal inflammation (36). *P. bivia*, a species associated with bacterial vaginosis, can facilitate the breakdown of the mucin layer of the vaginal epithelium to promote coinfection with other pathogens (37). *P. bivia* from the foreskin was found to increase cytokine production, recruit HIV-susceptible CD4^+^T cells, and was associated with HIV acquisition (38).

Meanwhile, various species were found to be enricheded in the SIRS(−) group, their beneficial role in human health. *S. paucimobilis* has the strong ability to generate chondroitinase, and this enzyme could degrade chondroitin sulfate A to unsaturated disaccharide, which attenuated damage and accelerated repair of injured myocardial cells to exert cardioprotective effects (39). *S. paucimobilis* also degrades ferulic acid to vanillin, a compound with anti-oxidant and anti-inflammatory capacities by scavenging reactive oxygen species and reducing inflammatory cytokines (40, 41). *A. johnsonii* was found to be more prevalent in the sinonasal microbiome of healthy controls compared with chronic rhinosinusitis patients and was associated with postoperative symptom improvement (42). *C. accolens* is increasingly considered a non-pathogenic bacterium that can protect against harmful microbes. Bomar et al. found that *C. accolens* expressed a lipase which cleaves host skin surface triacylglycerols into anti-microbial free fatty acids that inhibit growth of *Streptococcus pneumoniae* (43). *C. accolens* can inhibit the growth of *Staphylococcus aureus* and reduce the mucosal barrier damage caused by *S. aureus* to prevent its invasion (44). *C. accolens* was also reported to serve as gatekeepers against SARS-CoV-2 infection (45).

The functional profiles of the urobiome between the two groups were also predicted. KEGG analysis revealed that isoflavonoid biosynthesis and flavonoid biosynthesis were enriched in the SIRS(−) group. Isoflavonoids and flavonoids are a diverse group of polyphenolic compounds that have gained attention for their potential health benefits, including anti-oxidant, anti-inflammatory, and anti-microbial effects (46, 47). Besides, staurosporine biosynthesis and tetracycline biosynthesis were enriched in the SIRS(−) group, suggesting that the microbes in the SIRS(−) group might produce more antibiotics to kill or inhibit harmful pathogens.

Based on our results and existing studies, we speculated that the renal pelvis urobiome dysbiosis constituted an important factor in post-PCNL SIRS. Pathogenic microbes and their virulence factors can translocate into the blood vessels through the damaged endothelium. Their dissemination can activate the immune system, trigger the release of inflammatory cytokines, and eventually lead to SIRS. Protective microbes can produce bioactive compounds with anti-oxidant, anti-inflammatory, and anti-microbial properties. They inhibit the colonization of pathogenic microbes to maintain a balanced urobiome. The imbalance characterized by the overgrowth of pathogenic microbes and depletion of protective microbes likely represents a pivotal step in post-PCNL SIRS. Through differential analysis and random forest, we screened six differential species to build the microbial prediction model, which outperformed the clinical prediction model. Importantly, the combination of six differential species and two clinical factors yielded the best discriminator between the two groups.

Some limitations of our study should be noted. First, this was a single‐center study with a relatively small sample size, limiting the generalizability of the results. Second, stone composition analysis was not systematically conducted for all patients in this cohort. Future studies should include stone composition analysis to further stratify SIRS risk. Third, although 2bRAD-M is a precise technique to profile the renal pelvis urobiome, an integrated multi-omics approach (metagenomics and metabolomics) will allow a deeper insight into the host‐microbial interactions. Fourth, the biological mechanisms of how differential species involved in post-PCNL SIRS remain unclear. It is necessary to conduct cell and animal experiments to investigate the causal relationship between the microbes and SIRS. Finally, we address how the identified urobiome characteristics can be applied to clinical practice. We can design specific primers targeting pathogenic or protective species with high predictive value and perform targeted quantitative PCR to rapidly achieve qualitative and semi-quantitative detection of these microbes in preoperative urine samples. Based on the test results combined with clinical indicators, patients can undergo precise risk assessment. For high-risk patients, doctors can develop personalized preventive strategies, such as enhanced postoperative care and monitoring, and the use of prophylactic antibiotics. However, before clinical application, multicenter large-scale sample validation is needed to confirm the generalizability of the microbial biomarkers and to determine threshold values for the microbial abundances used in diagnosis.

### Conclusions

Our study was the first to apply 2bRAD-M to explore the renal pelvis urobiome of kidney stone patients with or without post-PCNL SIRS. Renal pelvis urobiome dysbiosis was observed between the two groups, and a number of differential species were identified through multiple approaches. The microbial prediction model included six differential species (*Ralstonia *sp000620465, *S. paucimobilis*, *R. pickettii*, *P. puraquae*, *C. tsuruhatensis*, and *L. clevelandensis_A*) outperformed the clinical model included two clinical factors (AGR and operative time). Besides, the prediction model incorporating microbial and clinical data exhibited the best predictive ability. We hope this work opens new avenues for the early prediction of post-PCNL SIRS.

## Data Availability

Sequencing data from this study have been deposited in the GenBank Sequence Read Archive under accession number PRJNA1267986.

## SUPPLEMENTAL MATERIAL

Table S1 msystems.00780-25-s0001.docx Comparison of average relative abundance of pelvis urinary microbiome between SIRS(−) and SIRS(+) at the genus and species levels.

Table S2 msystems.00780-25-s0002.docx Comparison of average abundance of predicted functions between SIRS(−) and SIRS(+).

ASM does not own the copyrights to Supplemental Material that may be linked to, or accessed through, an article. The authors have granted ASM a non-exclusive, world-wide license to publish the Supplemental Material files. Please contact the corresponding author directly for reuse.

## References

[1] Türk C, Petřík A, Sarica K, Seitz C, Skolarikos A, Straub M, Knoll T. 2016. EAU guidelines on interventional treatment for urolithiasis. Eur Urol 69:475–482. doi:10.1016/j.eururo.2015.07.041 26344917

[2] Assimos D, Krambeck A, Miller NL, Monga M, Murad MH, Nelson CP, Pace KT, Pais VM, Pearle MS, Preminger GM, Razvi H, Shah O, Matlaga BR. 2016. Surgical management of stones: American Urological Association/Endourological Society Guideline, PART II. J Urol 196:1153–1160. doi:10.1016/j.juro.2016.05.090 27238616

[3] Wollin DA, Preminger GM. 2018. Percutaneous nephrolithotomy: complications and how to deal with them. Urolithiasis 46:87–97. doi:10.1007/s00240-017-1022-x 29149365

[4] Korets R, Graversen JA, Kates M, Mues AC, Gupta M. 2011. Post-percutaneous nephrolithotomy systemic inflammatory response: a prospective analysis of preoperative urine, renal pelvic urine and stone cultures. J Urol 186:1899–1903. doi:10.1016/j.juro.2011.06.064 21944106

[5] de la Rosette J, Assimos D, Desai M, Gutierrez J, Lingeman J, Scarpa R, Tefekli A, CROES PCNL Study Group. 2011. The clinical research office of the endourological society percutaneous nephrolithotomy global study: indications, complications, and outcomes in 5803 patients. J Endourol 25:11–17. doi:10.1089/end.2010.0424 21247286

[6] Whitehurst L, Jones P, Somani BK. 2019. Mortality from kidney stone disease (KSD) as reported in the literature over the last two decades: a systematic review. World J Urol 37:759–776. doi:10.1007/s00345-018-2424-2 30151599

[7] He Z, Tang F, Lei H, Chen Y, Zeng G. 2018. Risk factors for systemic inflammatory response syndrome after percutaneous nephrolithotomy. Prog Urol 28:582–587. doi:10.1016/j.purol.2018.06.006 30301521

[8] Chen L, Xu QQ, Li JX, Xiong LL, Wang XF, Huang XB. 2008. Systemic inflammatory response syndrome after percutaneous nephrolithotomy: an assessment of risk factors. Int J Urol 15:1025–1028. doi:10.1111/j.1442-2042.2008.02170.x 19120510

[9] Erdil T, Bostanci Y, Ozden E, Atac F, Yakupoglu YK, Yilmaz AF, Sarikaya S. 2013. Risk factors for systemic inflammatory response syndrome following percutaneous nephrolithotomy. Urolithiasis 41:395–401. doi:10.1007/s00240-013-0570-y 23712738

[10] Zhou G, Zhou Y, Chen R, Wang D, Zhou S, Zhong J, Zhao Y, Wan C, Yang B, Xu J, Geng E, Li G, Huang Y, Liu H, Liu J. 2022. The influencing factors of infectious complications after percutaneous nephrolithotomy: a systematic review and meta-analysis. Urolithiasis 51:17. doi:10.1007/s00240-022-01376-5 36515726 PMC9750925

[11] Whiteside SA, Razvi H, Dave S, Reid G, Burton JP. 2015. The microbiome of the urinary tract—a role beyond infection. Nat Rev Urol 12:81–90. doi:10.1038/nrurol.2014.361 25600098

[12] Lee HY, Lin CY, Juan YS, Wu WJ, Cho SY, Wu DC. 2025. The influence and relationship of dysbiosis in the urinary microbiota on patients with urolithiasis. Urolithiasis 53:53. doi:10.1007/s00240-025-01724-1 40105975

[13] Gao H, Lin J, Xiong F, Yu Z, Pan S, Huang Y. 2022. Urinary microbial and metabolomic profiles in kidney stone disease. Front Cell Infect Microbiol 12:953392. doi:10.3389/fcimb.2022.953392 36132987 PMC9484321

[14] Agudelo-Ochoa GM, Valdés-Duque BE, Giraldo-Giraldo NA, Jaillier-Ramírez AM, Giraldo-Villa A, Acevedo-Castaño I, Yepes-Molina MA, Barbosa-Barbosa J, Benítez-Paéz A. 2020. Gut microbiota profiles in critically ill patients, potential biomarkers and risk variables for sepsis. Gut Microbes 12:1707610. doi:10.1080/19490976.2019.1707610 31924126 PMC7524144

[15] Liu W, Cheng M, Li J, Zhang P, Fan H, Hu Q, Han M, Su L, He H, Tong Y, Ning K, Long Y. 2020. Classification of the gut microbiota of patients in intensive care units during development of sepsis and septic shock. Genomics Proteomics Bioinformatics 18:696–707. doi:10.1016/j.gpb.2020.06.011 33607294 PMC8377022

[16] Levy MM, Fink MP, Marshall JC, Abraham E, Angus D, Cook D, Cohen J, Opal SM, Vincent J-L, Ramsay G. 2003. 2001 SCCM/ESICM/ACCP/ATS/SIS international sepsis definitions conference. Crit Care Med 31:1250–1256. doi:10.1097/01.CCM.0000050454.01978.3B 12682500

[17] Sun Z, Huang S, Zhu P, Tzehau L, Zhao H, Lv J, Zhang R, Zhou L, Niu Q, Wang X, Zhang M, Jing G, Bao Z, Liu J, Wang S, Xu J. 2022. Species-resolved sequencing of low-biomass or degraded microbiomes using 2bRAD-M. Genome Biol 23:36. doi:10.1186/s13059-021-02576-9 35078506 PMC8789378

[18] Dixon P. 2003. VEGAN, a package of R functions for community ecology. J Veg Sci 14:927–930. doi:10.1111/j.1654-1103.2003.tb02228.x

[19] Segata N, Izard J, Waldron L, Gevers D, Miropolsky L, Garrett WS, Huttenhower C. 2011. Metagenomic biomarker discovery and explanation. Genome Biol 12:R60. doi:10.1186/gb-2011-12-6-r60 21702898 PMC3218848

[20] Mallick H, Rahnavard A, McIver LJ, Ma S, Zhang Y, Nguyen LH, Tickle TL, Weingart G, Ren B, Schwager EH, Chatterjee S, Thompson KN, Wilkinson JE, Subramanian A, Lu Y, Waldron L, Paulson JN, Franzosa EA, Bravo HC, Huttenhower C. 2021. Multivariable association discovery in population-scale meta-omics studies. PLoS Comput Biol 17:e1009442. doi:10.1371/journal.pcbi.1009442 34784344 PMC8714082

[21] De Cáceres M, Legendre P, Moretti M. 2010. Improving indicator species analysis by combining groups of sites. Oikos 119:1674–1684. doi:10.1111/j.1600-0706.2010.18334.x

[22] Liaw A, Wiener M. 2002. Classification and regression by randomForest. R news 2:18–22. https://cran.r-project.org/doc/Rnews/Rnews_2002-3.pdf.

[23] Douglas GM, Maffei VJ, Zaneveld J, Yurgel SN, Brown JR, Taylor CM, Huttenhower C, Langille MGI. 2019. PICRUSt2: an improved and customizable approach for metagenome inference. bioRxiv. 10.1101/672295

[24] Wang Q, Jiang K, Chen X, Zeng G, Sun F. 2022. The predictive value of preoperative albumin-globulin ratio for systemic inflammatory response syndrome after percutaneous nephrolithotomy. Int J Gen Med 15:7407–7415. doi:10.2147/IJGM.S379741 36172085 PMC9512289

[25] McMillan DC, Watson WS, O’Gorman P, Preston T, Scott HR, McArdle CS. 2001. Albumin concentrations are primarily determined by the body cell mass and the systemic inflammatory response in cancer patients with weight loss. Nutr Cancer 39:210–213. doi:10.1207/S15327914nc392_8 11759282

[26] Morales F, Montserrat-de la Paz S, Leon MJ, Rivero-Pino F. 2023. Effects of malnutrition on the immune system and infection and the role of nutritional strategies regarding improvements in children’s health status: a literature review. Nutrients 16:1. doi:10.3390/nu16010001 38201831 PMC10780435

[27] Zhong W, Zeng G, Wu K, Li X, Chen W, Yang H. 2008. Does a smaller tract in percutaneous nephrolithotomy contribute to high renal pelvic pressure and postoperative fever? J Endourol 22:2147–2151. doi:10.1089/end.2008.0001 18811571

[28] Du B, Shen N, Tao Y, Sun S, Zhang F, Ren H, Cao Q, Mo X. 2021. Analysis of gut microbiota alteration and application as an auxiliary prognostic marker for sepsis in children: a pilot study. Transl Pediatr 10:1647–1657. doi:10.21037/tp-21-51 34295779 PMC8261590

[29] Liu Z, Li N, Fang H, Chen X, Guo Y, Gong S, Niu M, Zhou H, Jiang Y, Chang P, Chen P. 2019. Enteric dysbiosis is associated with sepsis in patients. FASEB J 33:12299–12310. doi:10.1096/fj.201900398RR 31465241 PMC6902702

[30] Rasmussen M. 2016. Aerococcus: an increasingly acknowledged human pathogen. Clin Microbiol Infect 22:22–27. doi:10.1016/j.cmi.2015.09.026 26454061

[31] Ma X, Kim J-K, Shin Y-J, Park H-S, Lee D-Y, Yim S-V, Kim D-H. 2024. Lipopolysaccharide-producing Veillonella infantium and Escherichia fergusonii cause vagus nerve-mediated cognitive impairment in mice. Brain Behav Immun 118:136–148. doi:10.1016/j.bbi.2024.02.031 38428648

[32] Izquierdo M, Lopez J, Gallardo P, Vidal RM, Ossa JC, Farfan MJ. 2022. Bacteria from gut microbiota associated with diarrheal infections in children promote virulence of Shiga toxin-producing and enteroaggregative Escherichia coli pathotypes. Front Cell Infect Microbiol 12:867205. doi:10.3389/fcimb.2022.867205 36017363 PMC9396624

[33] Kim D, Kim Y-G, Seo S-U, Kim D-J, Kamada N, Prescott D, Chamaillard M, Philpott DJ, Rosenstiel P, Inohara N, Núñez G. 2016. Nod2-mediated recognition of the microbiota is critical for mucosal adjuvant activity of cholera toxin. Nat Med 22:524–530. doi:10.1038/nm.4075 27064448 PMC4860092

[34] Liu X, Xu B, Xu X, Wang Z, Luo Y, Gao Y, Ling S, Wang A, Zhou Y, Wang X, Leng SX, Li W, Yao X. 2023. Attenuation of allergen-specific immunotherapy for atopic dermatitis by ectopic colonization of Brevundimonas vesicularis in the intestine. Cell Rep Med 4:101340. doi:10.1016/j.xcrm.2023.101340 38118418 PMC10772585

[35] Tsay J-CJ, Wu BG, Sulaiman I, Gershner K, Schluger R, Li Y, Yie T-A, Meyn P, Olsen E, Perez L. et al.. 2021. Lower airway dysbiosis affects lung cancer progression. Cancer Discov 11:293–307. doi:10.1158/2159-8290.CD-20-0263 33177060 PMC7858243

[36] Zhan Z, Liu W, Pan L, Bao Y, Yan Z, Hong L. 2022. Overabundance of Veillonella parvula promotes intestinal inflammation by activating macrophages via LPS-TLR4 pathway. Cell Death Discov 8:251. doi:10.1038/s41420-022-01015-3 35523778 PMC9076897

[37] Muzny CA, Taylor CM, Swords WE, Tamhane A, Chattopadhyay D, Cerca N, Schwebke JR. 2019. An updated conceptual model on the pathogenesis of bacterial vaginosis. J Infect Dis 220:1399–1405. doi:10.1093/infdis/jiz342 31369673 PMC6761952

[38] Prodger JL, Abraham AG, Tobian AA, Park DE, Aziz M, Roach K, Gray RH, Buchanan L, Kigozi G, Galiwango RM, Ssekasanvu J, Nnamutete J, Kagaayi J, Kaul R, Liu CM. 2021. Penile bacteria associated with HIV seroconversion, inflammation, and immune cells. JCI Insight 6:e147363. doi:10.1172/jci.insight.147363 33884964 PMC8119186

[39] Fu J, Jiang Z, Chang J, Han B, Liu W, Peng Y. 2018. Purification, characterization of chondroitinase ABC from Sphingomonas paucimobilis and in vitro cardiocytoprotection of the enzymatically degraded CS-A. Int J Biol Macromol 115:737–745. doi:10.1016/j.ijbiomac.2018.04.117 29702169

[40] Masai E, Harada K, Peng X, Kitayama H, Katayama Y, Fukuda M. 2002. Cloning and characterization of the ferulic acid catabolic genes of Sphingomonas paucimobilis SYK-6. Appl Environ Microbiol 68:4416–4424. doi:10.1128/AEM.68.9.4416-4424.2002 12200295 PMC124110

[41] Tripathi AS, Awasthi S, Maurya RK, Yasir M, Mohapatra L, Srivastav V. 2022. Protective effect of vanillin on the management of cecal ligation and puncture induced sepsis rat model. Microb Pathog 165:105493. doi:10.1016/j.micpath.2022.105493 35307600

[42] Cleland EJ, Bassiouni A, Vreugde S, Wormald PJ. 2016. The bacterial microbiome in chronic rhinosinusitis: richness, diversity, postoperative changes, and patient outcomes. Am J Rhinol Allergy 30:37–43. doi:10.2500/ajra.2016.30.4261 26867528

[43] Bomar L, Brugger SD, Yost BH, Davies SS, Lemon KP. 2016. Corynebacterium accolens releases antipneumococcal free fatty acids from human nostril and skin surface triacylglycerols. mBio 7:e01725-15. doi:10.1128/mBio.01725-15 26733066 PMC4725001

[44] Huang S, Hon K, Bennett C, Hu H, Menberu M, Wormald P-J, Zhao Y, Vreugde S, Liu S. 2022. Corynebacterium accolens inhibits Staphylococcus aureus induced mucosal barrier disruption. Front Microbiol 13:984741. doi:10.3389/fmicb.2022.984741 36187946 PMC9515799

[45] Szabo D, Ostorhazi E, Stercz B, Makra N, Penzes K, Kristof K, Antal I, Rethelyi JM, Zsigmond RI, Birtalan E, Merkely B, Tamas L. 2023. Specific nasopharyngeal Corynebacterium strains serve as gatekeepers against SARS-CoV-2 infection. Geroscience 45:2927–2938. doi:10.1007/s11357-023-00850-1 37338780 PMC10643471

[46] Zhang L, Ravipati AS, Koyyalamudi SR, Jeong SC, Reddy N, Smith PT, Bartlett J, Shanmugam K, Münch G, Wu MJ. 2011. Antioxidant and anti-inflammatory activities of selected medicinal plants containing phenolic and flavonoid compounds. J Agric Food Chem 59:12361–12367. doi:10.1021/jf203146e 22023309

[47] Lin H, Hu J, Mei F, Zhang Y, Ma Y, Chen Q, Wang C, Fu J, Yang M, Wen Z, Wang X, Qi J, Han H, Yang R, Yang Y. 2022. Anti-microbial efficacy, mechanisms and druggability evaluation of the natural flavonoids. J Appl Microbiol 133:1975–1988. doi:10.1111/jam.15705 35801665

