# Supplementary material for: Renal pelvis urobiome dysbiosis is associated with postoperative systemic inflammatory response syndrome after percutaneous nephrolithotomy
Source: mSystems. 2025 Aug 15;10(9):e00780-25. doi: 10.1128/msystems.00780-25 (PMC12455945; doi:10.1128/msystems.00780-25)
Supplement: Table S1 — Comparison of average relative abundance of pelvis urinary microbiome between SIRS(−) and SIRS(+) at the genus and species levels. [file msystems.00780-25-s0001.docx]

Table S1. Comparison of average relative abundance of pelvis urinary microbiome between SIRS(-) and SIRS(+) at the genus and species levels

| Taxa | | Average relative abundance (%) | | |
| --- | --- | --- | --- | --- |
|  |  | SIRS(-) | SIRS(+) | P value |
| Genus | Pelomonas | 1.565812757 | 0.239078159 | 0.000 |
|  | Burkholderia | 1.671421973 | 0.773645716 | 0.000 |
|  | Acinetobacter | 2.482434986 | 0.733210326 | 0.001 |
|  | Lawsonella | 1.343853118 | 0.47848836 | 0.001 |
|  | Paracoccus | 0.527246735 | 0.121395987 | 0.002 |
|  | Sphingomonas | 0.900964021 | 0.486412915 | 0.003 |
|  | Cutibacterium | 0.504530703 | 0.161526897 | 0.004 |
|  | Peptoniphilus_C | 0 | 0.031061134 | 0.004 |
|  | Staphylococcus_A | 0 | 0.011786662 | 0.004 |
|  | Gordonia | 0.897748366 | 0.450725168 | 0.004 |
|  | Geobacillus | 3.587462204 | 0.764884275 | 0.005 |
|  | Pseudonocardia | 0.251179883 | 0.118704551 | 0.008 |
|  | Corynebacterium | 0.931952442 | 0.405870805 | 0.010 |
|  | Methylobacterium | 0.158314956 | 0.177287403 | 0.010 |
|  | Saccharopolyspora | 0.043483846 | 0.007809887 | 0.011 |
|  | Staphylococcus | 1.144400617 | 0.151245472 | 0.016 |
|  | Perlucidibaca | 0.955700818 | 0.52928345 | 0.017 |
|  | Lactobacillus | 0.01303438 | 2.450204778 | 0.020 |
|  | Cellvibrio | 0.010979842 | 0 | 0.021 |
|  | Saccharomonospora | 0.006943806 | 0 | 0.021 |
|  | Paraburkholderia | 0.493602246 | 0.32962225 | 0.022 |
|  | Brevibacillus_D | 0.013715663 | 0 | 0.026 |
|  | Pseudomonas | 0.63106464 | 0.39070812 | 0.028 |
|  | Serinicoccus | 0.232007105 | 0.030031858 | 0.029 |
|  | Nocardioides | 0.083826378 | 0.017178736 | 0.032 |
|  | Bordetella_B | 0.17104223 | 0.104092816 | 0.036 |
|  | Ralstonia | 26.62753914 | 18.72401483 | 0.036 |
|  | Kocuria | 0.475161575 | 0.162733268 | 0.037 |
|  | Aquabacterium | 0.050424731 | 0.165056766 | 0.038 |
|  | Tersicoccus | 7.09032E-05 | 0.010351671 | 0.039 |
|  | Citrobacter | 0.004588409 | 0.000100227 | 0.042 |
|  | 13-2-20CM-66-19 | 0 | 8.9258E-07 | 0.044 |
|  | ALPHA2B | 0 | 2.12109E-05 | 0.044 |
|  | Actinobaculum | 0 | 0.078184853 | 0.044 |
|  | Alloscardovia | 0 | 0.000149558 | 0.044 |
|  | Antricoccus | 0 | 2.64911E-05 | 0.044 |
|  | Bacillus_J | 0 | 0.028272664 | 0.044 |
|  | Blautia_A | 0 | 0.007004622 | 0.044 |
|  | Bordetella_C | 0 | 6.00369E-05 | 0.044 |
|  | Bowdeniella | 0 | 9.08931E-05 | 0.044 |
|  | Brucella | 0 | 4.3428E-05 | 0.044 |
|  | Campylobacter_B | 0 | 0.032582884 | 0.044 |
|  | Cohnella | 0 | 1.87409E-05 | 0.044 |
|  | Conyzicola | 0 | 3.82405E-05 | 0.044 |
|  | Dialister_B | 0 | 0.00234483 | 0.044 |
|  | Dyadobacter | 0 | 0.004722873 | 0.044 |
|  | Eikenella | 0 | 0.001517239 | 0.044 |
|  | Enterococcus_E | 0 | 0.038518462 | 0.044 |
|  | Fastidiosipila | 0 | 0.006210832 | 0.044 |
|  | Franconibacter | 0 | 4.6348E-05 | 0.044 |
|  | Gandjariella | 0 | 1.01887E-05 | 0.044 |
|  | Gemmobacter_B | 0 | 5.01371E-05 | 0.044 |
|  | Gryllotalpicola | 0 | 7.10111E-05 | 0.044 |
|  | Herbiconiux | 0 | 0.000142299 | 0.044 |
|  | Im94 | 0 | 2.94824E-05 | 0.044 |
|  | Kaistia | 0 | 1.25554E-05 | 0.044 |
|  | Klebsiella_A | 0 | 0.005132596 | 0.044 |
|  | Lacisediminihabitans | 0 | 2.7441E-05 | 0.044 |
|  | Lysinimonas_A | 0 | 4.81357E-05 | 0.044 |
|  | M3007 | 0 | 0.006865003 | 0.044 |
|  | MB11C04 | 0 | 0.003900777 | 0.044 |
|  | Mageeibacillus | 0 | 3.61989E-06 | 0.044 |
|  | Marisediminicola | 0 | 2.85707E-05 | 0.044 |
|  | Meiothermus_B | 0 | 2.09712E-05 | 0.044 |
|  | Methylophilus | 0 | 0.312249484 | 0.044 |
|  | Methylotenera_A | 0 | 0.00159227 | 0.044 |
|  | Microterricola | 0 | 2.52825E-05 | 0.044 |
|  | Mitsuokella | 0 | 8.57338E-06 | 0.044 |
|  | Modestobacter | 0 | 5.8269E-05 | 0.044 |
|  | Mumia | 0 | 0.001363318 | 0.044 |
|  | Paenochrobactrum | 0 | 0.000133509 | 0.044 |
|  | Peptococcus | 0 | 0.024143378 | 0.044 |
|  | Peptoniphilus_B | 0 | 0.002193989 | 0.044 |
|  | Priestia | 0 | 0.000167737 | 0.044 |
|  | Protaetiibacter | 0 | 4.06987E-05 | 0.044 |
|  | Pseudaminobacter | 0 | 4.31074E-05 | 0.044 |
|  | Pyramidobacter | 0 | 8.93723E-06 | 0.044 |
|  | QEVD01 | 0 | 2.14421E-05 | 0.044 |
|  | Rhizorhabdus | 0 | 0.009405834 | 0.044 |
|  | Rhodovarius | 0 | 0.010237153 | 0.044 |
|  | Scardovia | 0 | 0.006219987 | 0.044 |
|  | Sphingosinicella | 0 | 0.001570245 | 0.044 |
|  | Sutterella | 0 | 0.005970276 | 0.044 |
|  | Tagaea | 0 | 0.000972614 | 0.044 |
|  | Tatumella | 0 | 0.002901524 | 0.044 |
|  | Thermicanus | 0 | 0.002980375 | 0.044 |
|  | Treponema_D | 0 | 0.001644387 | 0.044 |
|  | UBA2030 | 0 | 0.02761059 | 0.044 |
|  | Varibaculum | 0 | 0.063187115 | 0.044 |
|  | Vibrio | 0 | 0.011668638 | 0.044 |
|  | Yokenella | 0 | 3.0193E-05 | 0.044 |
|  | ZYF759 | 0 | 3.69672E-05 | 0.044 |
|  | Zhihengliuella | 0 | 6.79553E-05 | 0.044 |
|  | Rhodococcus | 0.33434865 | 0.156197542 | 0.045 |
|  | Pauljensenia | 0.488075074 | 0.240197218 | 0.046 |
|  | Streptomyces | 0.033066117 | 0.003501017 | 0.048 |
|  | Methyloversatilis | 0.00270746 | 0.031002339 | 0.048 |
| Species | Sphingomonas_paucimobilis | 0.494687028 | 0.193116139 | 0.000 |
|  | Pelomonas_puraquae | 1.487479778 | 0.238642014 | 0.000 |
|  | Escherichia_sp001660175 | 0 | 8.37169E-05 | 0.000 |
|  | Acinetobacter_guillouiae | 0.537353134 | 0.053845222 | 0.001 |
|  | Lawsonella_clevelandensis_A | 1.343853118 | 0.478341111 | 0.001 |
|  | Pelomonas_sp003963075 | 0.062634937 | 3.96689E-05 | 0.001 |
|  | Acinetobacter_johnsonii | 1.162154686 | 0.470502757 | 0.003 |
|  | Paraburkholderia_ferrariae | 0.034797257 | 0.010409431 | 0.003 |
|  | Escherichia_fergusonii | 0 | 0.000163834 | 0.004 |
|  | Microbacterium_sp000383475 | 0 | 0.004926742 | 0.004 |
|  | Peptoniphilus_C_coxii | 0 | 0.031061134 | 0.004 |
|  | Rhodococcus_ruber | 0 | 0.005054519 | 0.004 |
|  | Staphylococcus_A_sciuri | 0 | 0.011786662 | 0.004 |
|  | Geobacillus_thermoleovorans | 3.437051358 | 0.764884275 | 0.005 |
|  | Cutibacterium_granulosum | 0.46349832 | 0.157418854 | 0.007 |
|  | Aquabacterium_parvum | 0.043070006 | 0.005018397 | 0.008 |
|  | Pseudonocardia_ammonioxydans | 0.227270472 | 0.112806409 | 0.010 |
|  | Ralstonia_pickettii | 5.894606646 | 3.494152491 | 0.010 |
|  | Corynebacterium_accolens | 0.354276247 | 0.106820578 | 0.010 |
|  | Burkholderia_ubonensis | 0.27184265 | 0.128353722 | 0.011 |
|  | Microbacterium_lacticum | 0.12971828 | 0.018376148 | 0.012 |
|  | Ralstonia_sp000801955 | 0.221604889 | 0.099933127 | 0.013 |
|  | Pseudomonas_E_carnis | 0.561318547 | 0.260162385 | 0.017 |
|  | Janibacter_anophelis | 0.152010097 | 0.047526547 | 0.017 |
|  | Perlucidibaca_sp002943415 | 0.95470969 | 0.52928345 | 0.018 |
|  | Escherichia_albertii | 3.66924E-05 | 0.000257681 | 0.020 |
|  | Kocuria_palustris | 0.296023128 | 0.086735002 | 0.021 |
|  | Cellvibrio_sp008806975 | 0.010979842 | 0 | 0.021 |
|  | Comamonas_koreensis | 0.000211768 | 0.001175156 | 0.023 |
|  | Escherichia_sp005843885 | 5.61211E-05 | 0.000226528 | 0.023 |
|  | Ralstonia_insidiosa | 0.330265197 | 0.202872635 | 0.024 |
|  | Brevibacillus_D_fluminis | 0.013715663 | 0 | 0.026 |
|  | Burkholderia_vietnamiensis | 0.198346109 | 0.075410303 | 0.026 |
|  | Sphingobium_yanoikuyae | 0.111284731 | 0.059849681 | 0.026 |
|  | Pseudomonas_aeruginosa | 0.63106464 | 0.39070812 | 0.028 |
|  | Gordonia_jacobaea | 0.029765654 | 0.01412621 | 0.029 |
|  | Rhodococcus_qingshengii | 0.277853964 | 0.122046166 | 0.034 |
|  | Serinicoccus_profundi | 0.229609002 | 0.029940812 | 0.035 |
|  | Bordetella_B_ansorpii_B | 0.167339602 | 0.104092816 | 0.037 |
|  | Leucobacter_sp900163635 | 2.40495E-05 | 0.00091041 | 0.037 |
|  | Stenotrophomonas_maltophilia | 5.86535E-05 | 0.002334596 | 0.037 |
|  | Burkholderia_oklahomensis | 0.071746515 | 0.023312567 | 0.037 |
|  | Saccharomonospora_isguenensis | 0.005882786 | 0 | 0.038 |
|  | Bosea_sp900156025 | 9.21406E-05 | 0.000639676 | 0.039 |
|  | Microbacterium_sp011046485 | 3.69564E-05 | 0.004407246 | 0.039 |
|  | Capnocytophaga_granulosa | 0.002871533 | 0.005776051 | 0.042 |
|  | Paracoccus_sp009674885 | 4.95296E-05 | 0.0001144 | 0.042 |
|  | Prevotella_sp000467895 | 0.002733698 | 0.005018658 | 0.042 |
|  | Stenotrophomonas_maltophilia_AL | 0.015993446 | 0.000192527 | 0.042 |
|  | Burkholderia_sp000687455 | 0.105740556 | 0.037049996 | 0.043 |
|  | 13-2-20CM-66-19_sp003136935 | 0 | 8.9258E-07 | 0.044 |
|  | ALPHA2B_sp005503065 | 0 | 2.12109E-05 | 0.044 |
|  | Acidovorax_soli_A | 0 | 0.051100919 | 0.044 |
|  | Acidovorax_sp003208485 | 0 | 0.008855658 | 0.044 |
|  | Acidovorax_sp003852545 | 0 | 0.001714897 | 0.044 |
|  | Acidovorax_sp005405905 | 0 | 0.005245225 | 0.044 |
|  | Acidovorax_sp013408765 | 0 | 0.036192634 | 0.044 |
|  | Actinobaculum_massiliense | 0 | 0.078184853 | 0.044 |
|  | Actinotignum_sanguinis | 0 | 0.004095909 | 0.044 |
|  | Actinotignum_schaalii | 0 | 0.016494105 | 0.044 |
|  | Aerococcus_urinae_C | 0 | 0.011195571 | 0.044 |
|  | Aerococcus_urinae_D | 0 | 0.381710681 | 0.044 |
|  | Aeromicrobium_sp000471045 | 0 | 7.51801E-05 | 0.044 |
|  | Aeromicrobium_sp002174305 | 0 | 2.71589E-05 | 0.044 |
|  | Aeromonas_sanarellii | 0 | 0.002577966 | 0.044 |
|  | Agrococcus_sp005484985 | 0 | 6.96725E-05 | 0.044 |
|  | Agromyces_italicus | 0 | 2.42256E-05 | 0.044 |
|  | Alcaligenes_phenolicus | 0 | 0.000111018 | 0.044 |
|  | Alloscardovia_omnicolens | 0 | 0.000149558 | 0.044 |
|  | Amaricoccus_macauensis | 0 | 5.80438E-05 | 0.044 |
|  | Aminobacter_niigataensis | 0 | 6.56717E-05 | 0.044 |
|  | Anaerococcus_vaginalis_B | 0 | 0.029922566 | 0.044 |
|  | Antricoccus_suffuscus | 0 | 2.64911E-05 | 0.044 |
|  | Aquabacterium_commune | 0 | 0.023699235 | 0.044 |
|  | Aquabacterium_sp004310865 | 0 | 3.57361E-05 | 0.044 |
|  | Aquabacterium_sp903894125 | 0 | 0.004599896 | 0.044 |
|  | Aspergillus_terreus | 0 | 0.001309954 | 0.044 |
|  | Bacillus_J_thermoamylovorans | 0 | 0.028272664 | 0.044 |
|  | Bacteroides_sp900552405 | 0 | 0.000516317 | 0.044 |
|  | Bacteroides_stercorirosoris | 0 | 0.000101009 | 0.044 |
|  | Bacteroides_uniformis | 0 | 0.017061005 | 0.044 |
|  | Bifidobacterium_sp003585735 | 0 | 2.06468E-05 | 0.044 |
|  | Bifidobacterium_vaginale_D | 0 | 0.002636299 | 0.044 |
|  | Bifidobacterium_vaginale_G | 0 | 0.000230612 | 0.044 |
|  | Blastococcus_sp003319185 | 0 | 1.879E-05 | 0.044 |
|  | Blautia_A_massiliensis | 0 | 0.007004622 | 0.044 |
|  | Bordetella_C_sp002261215 | 0 | 6.00369E-05 | 0.044 |
|  | Bosea_sp005502805 | 0 | 0.00112705 | 0.044 |
|  | Bosea_sp008253865 | 0 | 0.000536116 | 0.044 |
|  | Bosea_sp011764485 | 0 | 8.62578E-05 | 0.044 |
|  | Bowdeniella_nasicola | 0 | 2.64749E-05 | 0.044 |
|  | Bowdeniella_nasicola_A | 0 | 6.44182E-05 | 0.044 |
|  | Brachybacterium_faecium | 0 | 0.000107707 | 0.044 |
|  | Brachybacterium_saurashtrense | 0 | 0.000111612 | 0.044 |
|  | Bradyrhizobium_guangzhouense | 0 | 0.000714123 | 0.044 |
|  | Brevundimonas_sp002157625 | 0 | 4.17722E-05 | 0.044 |
|  | Brucella_melitensis | 0 | 4.3428E-05 | 0.044 |
|  | Burkholderia_lata | 0 | 4.75043E-07 | 0.044 |
|  | Burkholderia_sp003635165 | 0 | 0.008361092 | 0.044 |
|  | Burkholderia_sp902833225 | 0 | 1.43562E-06 | 0.044 |
|  | CADCTG01_sp902805645 | 0 | 0.00198098 | 0.044 |
|  | CAG-873_sp009775535 | 0 | 0.000100485 | 0.044 |
|  | Caballeronia_insecticola | 0 | 2.24584E-05 | 0.044 |
|  | Campylobacter_B_ureolyticus | 0 | 0.025892124 | 0.044 |
|  | Campylobacter_B_ureolyticus_A | 0 | 0.00669076 | 0.044 |
|  | Caulobacter_sp000426025 | 0 | 4.82609E-05 | 0.044 |
|  | Caulobacter_sp004144935 | 0 | 4.08137E-05 | 0.044 |
|  | Centipeda_sp000468035 | 0 | 0.000107107 | 0.044 |
|  | Cereibacter_A_sphaeroides | 0 | 8.42726E-05 | 0.044 |
|  | Chlamydophila_avium | 0 | 0.000206056 | 0.044 |
|  | Citrobacter_freundii | 0 | 7.13856E-05 | 0.044 |
|  | Citrobacter_murliniae | 0 | 1.09432E-05 | 0.044 |
|  | Citrobacter_portucalensis_A | 0 | 1.78982E-05 | 0.044 |
|  | Cohnella_phaseoli | 0 | 1.87409E-05 | 0.044 |
|  | Comamonas_composti | 0 | 4.87735E-05 | 0.044 |
|  | Comamonas_piscis | 0 | 3.27064E-05 | 0.044 |
|  | Comamonas_testosteroni_B | 0 | 3.63801E-05 | 0.044 |
|  | Conyzicola_nivalis | 0 | 3.82405E-05 | 0.044 |
|  | Corynebacterium_frankenforstense | 0 | 0.003531117 | 0.044 |
|  | Corynebacterium_pollutisoli | 0 | 8.77107E-05 | 0.044 |
|  | Corynebacterium_sp001807265 | 0 | 0.002827541 | 0.044 |
|  | Corynebacterium_sp001875665 | 0 | 0.000407508 | 0.044 |
|  | Corynebacterium_sp001875725 | 0 | 0.002700703 | 0.044 |
|  | Corynebacterium_sp014490595 | 0 | 4.51211E-05 | 0.044 |
|  | Cumulibacter_manganitolerans | 0 | 9.54746E-05 | 0.044 |
|  | Curtobacterium_luteum_A | 0 | 0.002316184 | 0.044 |
|  | Curtobacterium_sp001864895 | 0 | 0.00070559 | 0.044 |
|  | DSM-21351_sp002250625 | 0 | 1.35662E-06 | 0.044 |
|  | Devosia_sp001425445 | 0 | 1.61377E-05 | 0.044 |
|  | Dialister_B_micraerophilus | 0 | 0.00234483 | 0.044 |
|  | Dyadobacter_sp001898145 | 0 | 0.004722873 | 0.044 |
|  | Eikenella_corrodens | 0 | 0.001517239 | 0.044 |
|  | Ensifer_sp900469595 | 0 | 1.93346E-05 | 0.044 |
|  | Enterococcus_E_cecorum | 0 | 0.038518462 | 0.044 |
|  | Epilithonimonas_sp003932955 | 0 | 7.56175E-05 | 0.044 |
|  | Erwinia_aphidicola | 0 | 5.76314E-06 | 0.044 |
|  | Erythrobacter_cryptus | 0 | 0.002117358 | 0.044 |
|  | Fastidiosipila_sanguinis | 0 | 0.006210832 | 0.044 |
|  | Franconibacter_helveticus | 0 | 4.6348E-05 | 0.044 |
|  | Frigoribacterium_faeni_A | 0 | 0.00683074 | 0.044 |
|  | Gandjariella_thermophila | 0 | 1.01887E-05 | 0.044 |
|  | Gemmobacter_B_sp002855575 | 0 | 5.01371E-05 | 0.044 |
|  | Granulicatella_sp001058355 | 0 | 8.92235E-05 | 0.044 |
|  | Gryllotalpicola_sp009780695 | 0 | 7.10111E-05 | 0.044 |
|  | Halolamina_pelagica | 0 | 3.46288E-05 | 0.044 |
|  | Herbiconiux_sp004297105 | 0 | 0.000142299 | 0.044 |
|  | Im94_sp009749525 | 0 | 2.94824E-05 | 0.044 |
|  | Kaistia_adipata | 0 | 1.25554E-05 | 0.044 |
|  | Klebsiella_A_michiganensis_B | 0 | 0.005132596 | 0.044 |
|  | Klebsiella_quasivariicola | 0 | 0.00046118 | 0.044 |
|  | Knoellia_remsis | 0 | 2.46063E-05 | 0.044 |
|  | Kocuria_atrinae | 0 | 0.000342606 | 0.044 |
|  | Lacisediminihabitans_profunda | 0 | 2.7441E-05 | 0.044 |
|  | Lactobacillus_crispatus | 0 | 0.31181548 | 0.044 |
|  | Lactobacillus_johnsonii | 0 | 0.000229763 | 0.044 |
|  | Lawsonella_clevelandensis | 0 | 0.000147249 | 0.044 |
|  | Leifsonia_aquatica_A | 0 | 4.6689E-05 | 0.044 |
|  | Leifsonia_sp002105485 | 0 | 2.50812E-05 | 0.044 |
|  | Leucobacter_sp000980875 | 0 | 0.000101622 | 0.044 |
|  | Leucobacter_sp002752355 | 0 | 3.88405E-05 | 0.044 |
|  | Leucobacter_sp014529985 | 0 | 2.92155E-05 | 0.044 |
|  | Leuconostoc_inhae | 0 | 0.000373234 | 0.044 |
|  | Luteimonas_sp002307375 | 0 | 2.62359E-05 | 0.044 |
|  | Lysinimonas_A_sp011620705 | 0 | 4.81357E-05 | 0.044 |
|  | Lysobacter_segetis | 0 | 4.01035E-05 | 0.044 |
|  | Lysobacter_sp004361065 | 0 | 1.88172E-05 | 0.044 |
|  | M3007_sp903905445 | 0 | 0.006865003 | 0.044 |
|  | MB11C04_sp002722545 | 0 | 0.003900777 | 0.044 |
|  | Mageeibacillus_indolicus | 0 | 3.61989E-06 | 0.044 |
|  | Malassezia_sympodialis | 0 | 2.34628E-05 | 0.044 |
|  | Marisediminicola_antarctica | 0 | 2.4184E-05 | 0.044 |
|  | Marisediminicola_sp014378715 | 0 | 4.38667E-06 | 0.044 |
|  | Massilia_oculi | 0 | 6.51401E-05 | 0.044 |
|  | Meiothermus_B_silvanus | 0 | 2.09712E-05 | 0.044 |
|  | Mesorhizobium_defluvii | 0 | 0.000151922 | 0.044 |
|  | Mesorhizobium_sp004791165 | 0 | 1.78713E-05 | 0.044 |
|  | Methylobacterium_sp001423085 | 0 | 5.25849E-05 | 0.044 |
|  | Methylophilus_medardicus | 0 | 0.012419722 | 0.044 |
|  | Methylophilus_methylotrophus | 0 | 0.091084935 | 0.044 |
|  | Methylophilus_sp001183865 | 0 | 0.074888636 | 0.044 |
|  | Methylophilus_sp001424665 | 0 | 0.005754297 | 0.044 |
|  | Methylophilus_sp008015755 | 0 | 0.128101894 | 0.044 |
|  | Methylotenera_A_sp002083635 | 0 | 0.00159227 | 0.044 |
|  | Methyloversatilis_universalis | 0 | 0.009972929 | 0.044 |
|  | Methyloversatilis_universalis_A | 0 | 1.17533E-05 | 0.044 |
|  | Microbacterium_A_agarici | 0 | 0.000232689 | 0.044 |
|  | Microbacterium_enclense | 0 | 4.11168E-05 | 0.044 |
|  | Microbacterium_enclense_A | 0 | 2.82888E-05 | 0.044 |
|  | Microbacterium_esteraromaticum_C | 0 | 7.7266E-05 | 0.044 |
|  | Microbacterium_gubbeenense | 0 | 1.77064E-05 | 0.044 |
|  | Microbacterium_hominis_B | 0 | 7.64084E-05 | 0.044 |
|  | Microbacterium_immunditiarum | 0 | 2.43921E-05 | 0.044 |
|  | Microbacterium_indicum | 0 | 6.91377E-05 | 0.044 |
|  | Microbacterium_invictum | 0 | 1.54802E-05 | 0.044 |
|  | Microbacterium_lacus | 0 | 0.000186392 | 0.044 |
|  | Microbacterium_mangrovi | 0 | 2.39739E-05 | 0.044 |
|  | Microbacterium_oleivorans | 0 | 1.72385E-05 | 0.044 |
|  | Microbacterium_oryzae | 0 | 0.000242931 | 0.044 |
|  | Microbacterium_phyllosphaerae | 0 | 3.23873E-05 | 0.044 |
|  | Microbacterium_protaetiae | 0 | 4.80075E-05 | 0.044 |
|  | Microbacterium_saccharophilum | 0 | 0.000323553 | 0.044 |
|  | Microbacterium_sp000411455 | 0 | 0.000111037 | 0.044 |
|  | Microbacterium_sp001049495 | 0 | 0.000105479 | 0.044 |
|  | Microbacterium_sp001314225 | 0 | 2.01768E-05 | 0.044 |
|  | Microbacterium_sp001423485 | 0 | 5.45667E-05 | 0.044 |
|  | Microbacterium_sp001427145 | 0 | 0.00010529 | 0.044 |
|  | Microbacterium_sp001428485 | 0 | 0.000885443 | 0.044 |
|  | Microbacterium_sp001639925 | 0 | 0.000210642 | 0.044 |
|  | Microbacterium_sp001897945 | 0 | 9.77547E-05 | 0.044 |
|  | Microbacterium_sp001898325 | 0 | 0.0001053 | 0.044 |
|  | Microbacterium_sp002245215 | 0 | 1.63327E-05 | 0.044 |
|  | Microbacterium_sp003075395 | 0 | 5.01341E-05 | 0.044 |
|  | Microbacterium_sp004366135 | 0 | 0.000104359 | 0.044 |
|  | Microbacterium_sp004794465 | 0 | 0.000206504 | 0.044 |
|  | Microbacterium_sp006715565 | 0 | 4.00526E-05 | 0.044 |
|  | Microbacterium_sp006715675 | 0 | 3.24563E-05 | 0.044 |
|  | Microbacterium_sp007667425 | 0 | 0.000531207 | 0.044 |
|  | Microbacterium_sp007828185 | 0 | 4.37115E-05 | 0.044 |
|  | Microbacterium_sp009649635 | 0 | 0.00012361 | 0.044 |
|  | Microbacterium_sp011326725 | 0 | 0.000114387 | 0.044 |
|  | Microbacterium_sp013409015 | 0 | 0.000202699 | 0.044 |
|  | Microbacterium_sp900156455 | 0 | 3.63894E-05 | 0.044 |
|  | Microbacterium_testaceum_F | 0 | 5.13617E-05 | 0.044 |
|  | Microbacterium_yannicii | 0 | 4.75259E-05 | 0.044 |
|  | Microcella_alkaliphila_A | 0 | 3.70834E-05 | 0.044 |
|  | Microterricola_sp000799285 | 0 | 2.52825E-05 | 0.044 |
|  | Mitsuokella_sp000469545 | 0 | 8.57338E-06 | 0.044 |
|  | Modestobacter_marinus_A | 0 | 5.8269E-05 | 0.044 |
|  | Mumia_xiangluensis | 0 | 0.001363318 | 0.044 |
|  | Mycobacterium_obuense | 0 | 0.004720723 | 0.044 |
|  | Neorhizobium_sp002500765 | 0 | 0.000129159 | 0.044 |
|  | Nocardia_nova_B | 0 | 0.000167164 | 0.044 |
|  | Novosphingobium_guangzhouense | 0 | 0.003210871 | 0.044 |
|  | Ochrobactrum_sp900470195 | 0 | 6.86715E-05 | 0.044 |
|  | Paenochrobactrum_gallinarii | 0 | 0.000133509 | 0.044 |
|  | Paraburkholderia_bannensis | 0 | 0.003089552 | 0.044 |
|  | Paraburkholderia_phenazinium_A | 0 | 8.2954E-06 | 0.044 |
|  | Paraburkholderia_sp900996235 | 0 | 0.004214683 | 0.044 |
|  | Paracoccus_homiensis | 0 | 6.20378E-05 | 0.044 |
|  | Paracoccus_ravus | 0 | 6.1652E-05 | 0.044 |
|  | Paracoccus_siganidrum | 0 | 0.00013159 | 0.044 |
|  | Paracoccus_sp002359815 | 0 | 5.53363E-05 | 0.044 |
|  | Paracoccus_thiocyanatus | 0 | 0.000129755 | 0.044 |
|  | Pararhizobium_sp003217095 | 0 | 2.67411E-05 | 0.044 |
|  | Pararhizobium_sp900067135 | 0 | 3.34936E-05 | 0.044 |
|  | Pauljensenia_sp900541895 | 0 | 0.000293789 | 0.044 |
|  | Pauljensenia_turicensis | 0 | 6.01208E-05 | 0.044 |
|  | Peptococcus_niger | 0 | 0.024143378 | 0.044 |
|  | Peptoniphilus_A_grossensis | 0 | 0.001964935 | 0.044 |
|  | Peptoniphilus_B_sp000478985 | 0 | 0.002193989 | 0.044 |
|  | Plantibacter_sp001423185 | 0 | 2.1252E-05 | 0.044 |
|  | Porphyromonas_A_somerae | 0 | 0.015016218 | 0.044 |
|  | Porphyromonas_asaccharolytica | 0 | 0.001483573 | 0.044 |
|  | Porphyromonas_sp900539155 | 0 | 0.000292532 | 0.044 |
|  | Porphyromonas_sp900546675 | 0 | 0.000212578 | 0.044 |
|  | Porphyromonas_sp900548415 | 0 | 0.004298855 | 0.044 |
|  | Prevotella_seregens | 0 | 0.012583208 | 0.044 |
|  | Prevotella_sp013333285 | 0 | 0.015593855 | 0.044 |
|  | Priestia_flexa | 0 | 0.000167737 | 0.044 |
|  | Propionimicrobium_sp900155645 | 0 | 7.30142E-05 | 0.044 |
|  | Protaetiibacter_sp014483895 | 0 | 4.06987E-05 | 0.044 |
|  | Pseudaminobacter_arsenicus | 0 | 4.31074E-05 | 0.044 |
|  | Pseudomonas_A_stutzeri_P | 0 | 1.45357E-05 | 0.044 |
|  | Pseudomonas_A_xanthomarina | 0 | 1.0996E-06 | 0.044 |
|  | Pseudomonas_E_abietaniphila | 0 | 5.49541E-05 | 0.044 |
|  | Pseudomonas_E_bohemica | 0 | 0.001576155 | 0.044 |
|  | Pseudomonas_E_chlororaphis_F | 0 | 0.001681781 | 0.044 |
|  | Pseudomonas_E_massiliensis | 0 | 3.84271E-05 | 0.044 |
|  | Pseudomonas_E_mendocina | 0 | 0.023330174 | 0.044 |
|  | Pseudoxanthomonas_A_kalamensis | 0 | 5.2968E-05 | 0.044 |
|  | Psychrobacter_sp002414005 | 0 | 0.000372471 | 0.044 |
|  | Puccinia_triticina | 0 | 0.003509436 | 0.044 |
|  | Pyramidobacter_piscolens | 0 | 8.93723E-06 | 0.044 |
|  | QEVD01_sp003576975 | 0 | 2.14421E-05 | 0.044 |
|  | Rhizorhabdus_sp004297635 | 0 | 0.009405834 | 0.044 |
|  | Rhodovarius_lipocyclicus | 0 | 0.010237153 | 0.044 |
|  | Rubrivivax_sp001725505 | 0 | 0.000400931 | 0.044 |
|  | SCN-69-89_sp008039575 | 0 | 1.25471E-05 | 0.044 |
|  | Saccharimonas_sp013333675 | 0 | 8.15722E-05 | 0.044 |
|  | Scardovia_wiggsiae | 0 | 0.006219987 | 0.044 |
|  | Shewanella_morhuae | 0 | 3.62951E-05 | 0.044 |
|  | Shinella_sp001713395 | 0 | 2.87824E-05 | 0.044 |
|  | Sphingobacterium_sp000938735 | 0 | 4.63404E-05 | 0.044 |
|  | Sphingobacterium_sp002734245 | 0 | 0.002015203 | 0.044 |
|  | Sphingobium_barthaii_A | 0 | 4.80721E-05 | 0.044 |
|  | Sphingomicrobium_rhizophila | 0 | 0.003599011 | 0.044 |
|  | Sphingomicrobium_sp902806285 | 0 | 7.33831E-05 | 0.044 |
|  | Sphingomonas_sp000251145 | 0 | 2.81273E-05 | 0.044 |
|  | Sphingomonas_sp002292295 | 0 | 8.73796E-06 | 0.044 |
|  | Sphingomonas_sp903884945 | 0 | 0.002149398 | 0.044 |
|  | Sphingopyxis_sp001468285 | 0 | 2.98454E-05 | 0.044 |
|  | Sphingopyxis_sp012035435 | 0 | 4.71542E-05 | 0.044 |
|  | Sphingosinicella_sp013911755 | 0 | 0.001570245 | 0.044 |
|  | Staphylococcus_saprophyticus | 0 | 0.000114384 | 0.044 |
|  | Stenotrophomonas_maltophilia_AN | 0 | 7.42499E-05 | 0.044 |
|  | Streptomyces_sp001984575 | 0 | 5.50017E-06 | 0.044 |
|  | Streptomyces_sp004193175 | 0 | 2.80193E-05 | 0.044 |
|  | Sutterella_sp900762445 | 0 | 0.005970276 | 0.044 |
|  | Tagaea_sp014444615 | 0 | 0.000972614 | 0.044 |
|  | Tatumella_citrea | 0 | 0.002901524 | 0.044 |
|  | Tersicoccus_phoenicis | 0 | 0.010285849 | 0.044 |
|  | Tersicoccus_sp001968825 | 0 | 6.5821E-05 | 0.044 |
|  | Thauera_propionica | 0 | 0.00094978 | 0.044 |
|  | Thermicanus_aegyptius | 0 | 0.002980375 | 0.044 |
|  | Thermomonas_fusca | 0 | 4.56174E-05 | 0.044 |
|  | Treponema_D_sp014334325 | 0 | 0.001644387 | 0.044 |
|  | Trichoderma_asperellum | 0 | 4.31257E-06 | 0.044 |
|  | UBA2030_sp002332755 | 0 | 0.02761059 | 0.044 |
|  | Varibaculum_massiliense | 0 | 0.063187115 | 0.044 |
|  | Veillonella_sp900757715 | 0 | 0.015989713 | 0.044 |
|  | Vibrio_fluvialis | 0 | 0.011668638 | 0.044 |
|  | Yokenella_regensburgei | 0 | 3.0193E-05 | 0.044 |
|  | ZYF759_sp012911015 | 0 | 3.69672E-05 | 0.044 |
|  | Zhihengliuella_salsuginis | 0 | 6.79553E-05 | 0.044 |
|  | Paracoccus_marinus | 0.111002039 | 0.019005939 | 0.045 |
|  | Pauljensenia_odontolytica | 0.091674353 | 0.069328048 | 0.046 |
|  | Ralstonia_sp000620465 | 15.15887812 | 11.12343742 | 0.046 |
|  | Janibacter_melonis | 0.052011176 | 0.011852428 | 0.048 |
|  | Gordonia_sputi | 0.062712875 | 0.124955315 | 0.049 |
|  | Caballeronia_udeis | 0.006928366 | 9.78639E-07 | 0.050 |
|  | Sphingomonas_paucimobilis | 0.494687028 | 0.193116139 | 0.000 |
|  | Pelomonas_puraquae | 1.487479778 | 0.238642014 | 0.000 |
|  | Escherichia_sp001660175 | 0 | 8.37169E-05 | 0.000 |
|  | Acinetobacter_guillouiae | 0.537353134 | 0.053845222 | 0.001 |
|  | Lawsonella_clevelandensis_A | 1.343853118 | 0.478341111 | 0.001 |
|  | Pelomonas_sp003963075 | 0.062634937 | 3.96689E-05 | 0.001 |
|  | Acinetobacter_johnsonii | 1.162154686 | 0.470502757 | 0.003 |
|  | Paraburkholderia_ferrariae | 0.034797257 | 0.010409431 | 0.003 |
|  | Escherichia_fergusonii | 0 | 0.000163834 | 0.004 |
|  | Microbacterium_sp000383475 | 0 | 0.004926742 | 0.004 |
|  | Peptoniphilus_C_coxii | 0 | 0.031061134 | 0.004 |
|  | Rhodococcus_ruber | 0 | 0.005054519 | 0.004 |
|  | Staphylococcus_A_sciuri | 0 | 0.011786662 | 0.004 |
|  | Geobacillus_thermoleovorans | 3.437051358 | 0.764884275 | 0.005 |
|  | Cutibacterium_granulosum | 0.46349832 | 0.157418854 | 0.007 |
|  | Aquabacterium_parvum | 0.043070006 | 0.005018397 | 0.008 |
|  | Pseudonocardia_ammonioxydans | 0.227270472 | 0.112806409 | 0.010 |
|  | Ralstonia_pickettii | 5.894606646 | 3.494152491 | 0.010 |
|  | Corynebacterium_accolens | 0.354276247 | 0.106820578 | 0.010 |
|  | Burkholderia_ubonensis | 0.27184265 | 0.128353722 | 0.011 |
|  | Microbacterium_lacticum | 0.12971828 | 0.018376148 | 0.012 |
|  | Ralstonia_sp000801955 | 0.221604889 | 0.099933127 | 0.013 |
|  | Pseudomonas_E_carnis | 0.561318547 | 0.260162385 | 0.017 |
|  | Janibacter_anophelis | 0.152010097 | 0.047526547 | 0.017 |
|  | Perlucidibaca_sp002943415 | 0.95470969 | 0.52928345 | 0.018 |
|  | Escherichia_albertii | 3.66924E-05 | 0.000257681 | 0.020 |
|  | Kocuria_palustris | 0.296023128 | 0.086735002 | 0.021 |
|  | Cellvibrio_sp008806975 | 0.010979842 | 0 | 0.021 |
|  | Comamonas_koreensis | 0.000211768 | 0.001175156 | 0.023 |
|  | Escherichia_sp005843885 | 5.61211E-05 | 0.000226528 | 0.023 |
|  | Ralstonia_insidiosa | 0.330265197 | 0.202872635 | 0.024 |
|  | Brevibacillus_D_fluminis | 0.013715663 | 0 | 0.026 |
|  | Burkholderia_vietnamiensis | 0.198346109 | 0.075410303 | 0.026 |
|  | Sphingobium_yanoikuyae | 0.111284731 | 0.059849681 | 0.026 |
|  | Pseudomonas_aeruginosa | 0.63106464 | 0.39070812 | 0.028 |
|  | Gordonia_jacobaea | 0.029765654 | 0.01412621 | 0.029 |
|  | Rhodococcus_qingshengii | 0.277853964 | 0.122046166 | 0.034 |
|  | Serinicoccus_profundi | 0.229609002 | 0.029940812 | 0.035 |
|  | Bordetella_B_ansorpii_B | 0.167339602 | 0.104092816 | 0.037 |
|  | Leucobacter_sp900163635 | 2.40495E-05 | 0.00091041 | 0.037 |
|  | Stenotrophomonas_maltophilia | 5.86535E-05 | 0.002334596 | 0.037 |
|  | Burkholderia_oklahomensis | 0.071746515 | 0.023312567 | 0.037 |
|  | Saccharomonospora_isguenensis | 0.005882786 | 0 | 0.038 |
|  | Bosea_sp900156025 | 9.21406E-05 | 0.000639676 | 0.039 |
|  | Microbacterium_sp011046485 | 3.69564E-05 | 0.004407246 | 0.039 |
|  | Capnocytophaga_granulosa | 0.002871533 | 0.005776051 | 0.042 |
|  | Paracoccus_sp009674885 | 4.95296E-05 | 0.0001144 | 0.042 |
|  | Prevotella_sp000467895 | 0.002733698 | 0.005018658 | 0.042 |
|  | Stenotrophomonas_maltophilia_AL | 0.015993446 | 0.000192527 | 0.042 |
|  | Burkholderia_sp000687455 | 0.105740556 | 0.037049996 | 0.043 |
|  | 13-2-20CM-66-19_sp003136935 | 0 | 8.9258E-07 | 0.044 |
|  | ALPHA2B_sp005503065 | 0 | 2.12109E-05 | 0.044 |
|  | Acidovorax_soli_A | 0 | 0.051100919 | 0.044 |
|  | Acidovorax_sp003208485 | 0 | 0.008855658 | 0.044 |
|  | Acidovorax_sp003852545 | 0 | 0.001714897 | 0.044 |
|  | Acidovorax_sp005405905 | 0 | 0.005245225 | 0.044 |
|  | Acidovorax_sp013408765 | 0 | 0.036192634 | 0.044 |
|  | Actinobaculum_massiliense | 0 | 0.078184853 | 0.044 |
|  | Actinotignum_sanguinis | 0 | 0.004095909 | 0.044 |
|  | Actinotignum_schaalii | 0 | 0.016494105 | 0.044 |
|  | Aerococcus_urinae_C | 0 | 0.011195571 | 0.044 |
|  | Aerococcus_urinae_D | 0 | 0.381710681 | 0.044 |
|  | Aeromicrobium_sp000471045 | 0 | 7.51801E-05 | 0.044 |
|  | Aeromicrobium_sp002174305 | 0 | 2.71589E-05 | 0.044 |
|  | Aeromonas_sanarellii | 0 | 0.002577966 | 0.044 |
|  | Agrococcus_sp005484985 | 0 | 6.96725E-05 | 0.044 |
|  | Agromyces_italicus | 0 | 2.42256E-05 | 0.044 |
|  | Alcaligenes_phenolicus | 0 | 0.000111018 | 0.044 |
|  | Alloscardovia_omnicolens | 0 | 0.000149558 | 0.044 |
|  | Amaricoccus_macauensis | 0 | 5.80438E-05 | 0.044 |
|  | Aminobacter_niigataensis | 0 | 6.56717E-05 | 0.044 |
|  | Anaerococcus_vaginalis_B | 0 | 0.029922566 | 0.044 |
|  | Antricoccus_suffuscus | 0 | 2.64911E-05 | 0.044 |
|  | Aquabacterium_commune | 0 | 0.023699235 | 0.044 |
|  | Aquabacterium_sp004310865 | 0 | 3.57361E-05 | 0.044 |
|  | Aquabacterium_sp903894125 | 0 | 0.004599896 | 0.044 |
|  | Aspergillus_terreus | 0 | 0.001309954 | 0.044 |
|  | Bacillus_J_thermoamylovorans | 0 | 0.028272664 | 0.044 |
|  | Bacteroides_sp900552405 | 0 | 0.000516317 | 0.044 |
|  | Bacteroides_stercorirosoris | 0 | 0.000101009 | 0.044 |
|  | Bacteroides_uniformis | 0 | 0.017061005 | 0.044 |
|  | Bifidobacterium_sp003585735 | 0 | 2.06468E-05 | 0.044 |
|  | Bifidobacterium_vaginale_D | 0 | 0.002636299 | 0.044 |
|  | Bifidobacterium_vaginale_G | 0 | 0.000230612 | 0.044 |
|  | Blastococcus_sp003319185 | 0 | 1.879E-05 | 0.044 |
|  | Blautia_A_massiliensis | 0 | 0.007004622 | 0.044 |
|  | Bordetella_C_sp002261215 | 0 | 6.00369E-05 | 0.044 |
|  | Bosea_sp005502805 | 0 | 0.00112705 | 0.044 |
|  | Bosea_sp008253865 | 0 | 0.000536116 | 0.044 |
|  | Bosea_sp011764485 | 0 | 8.62578E-05 | 0.044 |
|  | Bowdeniella_nasicola | 0 | 2.64749E-05 | 0.044 |
|  | Bowdeniella_nasicola_A | 0 | 6.44182E-05 | 0.044 |
|  | Brachybacterium_faecium | 0 | 0.000107707 | 0.044 |
|  | Brachybacterium_saurashtrense | 0 | 0.000111612 | 0.044 |
|  | Bradyrhizobium_guangzhouense | 0 | 0.000714123 | 0.044 |
|  | Brevundimonas_sp002157625 | 0 | 4.17722E-05 | 0.044 |
|  | Brucella_melitensis | 0 | 4.3428E-05 | 0.044 |
|  | Burkholderia_lata | 0 | 4.75043E-07 | 0.044 |
|  | Burkholderia_sp003635165 | 0 | 0.008361092 | 0.044 |
|  | Burkholderia_sp902833225 | 0 | 1.43562E-06 | 0.044 |
|  | CADCTG01_sp902805645 | 0 | 0.00198098 | 0.044 |
|  | CAG-873_sp009775535 | 0 | 0.000100485 | 0.044 |
|  | Caballeronia_insecticola | 0 | 2.24584E-05 | 0.044 |
|  | Campylobacter_B_ureolyticus | 0 | 0.025892124 | 0.044 |
|  | Campylobacter_B_ureolyticus_A | 0 | 0.00669076 | 0.044 |
|  | Caulobacter_sp000426025 | 0 | 4.82609E-05 | 0.044 |
|  | Caulobacter_sp004144935 | 0 | 4.08137E-05 | 0.044 |
|  | Centipeda_sp000468035 | 0 | 0.000107107 | 0.044 |
|  | Cereibacter_A_sphaeroides | 0 | 8.42726E-05 | 0.044 |
|  | Chlamydophila_avium | 0 | 0.000206056 | 0.044 |
|  | Citrobacter_freundii | 0 | 7.13856E-05 | 0.044 |
|  | Citrobacter_murliniae | 0 | 1.09432E-05 | 0.044 |
|  | Citrobacter_portucalensis_A | 0 | 1.78982E-05 | 0.044 |
|  | Cohnella_phaseoli | 0 | 1.87409E-05 | 0.044 |
|  | Comamonas_composti | 0 | 4.87735E-05 | 0.044 |
|  | Comamonas_piscis | 0 | 3.27064E-05 | 0.044 |
|  | Comamonas_testosteroni_B | 0 | 3.63801E-05 | 0.044 |
|  | Conyzicola_nivalis | 0 | 3.82405E-05 | 0.044 |
|  | Corynebacterium_frankenforstense | 0 | 0.003531117 | 0.044 |
|  | Corynebacterium_pollutisoli | 0 | 8.77107E-05 | 0.044 |
|  | Corynebacterium_sp001807265 | 0 | 0.002827541 | 0.044 |
|  | Corynebacterium_sp001875665 | 0 | 0.000407508 | 0.044 |
|  | Corynebacterium_sp001875725 | 0 | 0.002700703 | 0.044 |
|  | Corynebacterium_sp014490595 | 0 | 4.51211E-05 | 0.044 |
|  | Cumulibacter_manganitolerans | 0 | 9.54746E-05 | 0.044 |
|  | Curtobacterium_luteum_A | 0 | 0.002316184 | 0.044 |
|  | Curtobacterium_sp001864895 | 0 | 0.00070559 | 0.044 |
|  | DSM-21351_sp002250625 | 0 | 1.35662E-06 | 0.044 |
|  | Devosia_sp001425445 | 0 | 1.61377E-05 | 0.044 |
|  | Dialister_B_micraerophilus | 0 | 0.00234483 | 0.044 |
|  | Dyadobacter_sp001898145 | 0 | 0.004722873 | 0.044 |
|  | Eikenella_corrodens | 0 | 0.001517239 | 0.044 |
|  | Ensifer_sp900469595 | 0 | 1.93346E-05 | 0.044 |
|  | Enterococcus_E_cecorum | 0 | 0.038518462 | 0.044 |
|  | Epilithonimonas_sp003932955 | 0 | 7.56175E-05 | 0.044 |
|  | Erwinia_aphidicola | 0 | 5.76314E-06 | 0.044 |
|  | Erythrobacter_cryptus | 0 | 0.002117358 | 0.044 |
|  | Fastidiosipila_sanguinis | 0 | 0.006210832 | 0.044 |
|  | Franconibacter_helveticus | 0 | 4.6348E-05 | 0.044 |
|  | Frigoribacterium_faeni_A | 0 | 0.00683074 | 0.044 |
|  | Gandjariella_thermophila | 0 | 1.01887E-05 | 0.044 |
|  | Gemmobacter_B_sp002855575 | 0 | 5.01371E-05 | 0.044 |
|  | Granulicatella_sp001058355 | 0 | 8.92235E-05 | 0.044 |
|  | Gryllotalpicola_sp009780695 | 0 | 7.10111E-05 | 0.044 |
|  | Halolamina_pelagica | 0 | 3.46288E-05 | 0.044 |
|  | Herbiconiux_sp004297105 | 0 | 0.000142299 | 0.044 |
|  | Im94_sp009749525 | 0 | 2.94824E-05 | 0.044 |
|  | Kaistia_adipata | 0 | 1.25554E-05 | 0.044 |
|  | Klebsiella_A_michiganensis_B | 0 | 0.005132596 | 0.044 |
|  | Klebsiella_quasivariicola | 0 | 0.00046118 | 0.044 |
|  | Knoellia_remsis | 0 | 2.46063E-05 | 0.044 |
|  | Kocuria_atrinae | 0 | 0.000342606 | 0.044 |
|  | Lacisediminihabitans_profunda | 0 | 2.7441E-05 | 0.044 |
|  | Lactobacillus_crispatus | 0 | 0.31181548 | 0.044 |
|  | Lactobacillus_johnsonii | 0 | 0.000229763 | 0.044 |
|  | Lawsonella_clevelandensis | 0 | 0.000147249 | 0.044 |
|  | Leifsonia_aquatica_A | 0 | 4.6689E-05 | 0.044 |
|  | Leifsonia_sp002105485 | 0 | 2.50812E-05 | 0.044 |
|  | Leucobacter_sp000980875 | 0 | 0.000101622 | 0.044 |
|  | Leucobacter_sp002752355 | 0 | 3.88405E-05 | 0.044 |
|  | Leucobacter_sp014529985 | 0 | 2.92155E-05 | 0.044 |
|  | Leuconostoc_inhae | 0 | 0.000373234 | 0.044 |
|  | Luteimonas_sp002307375 | 0 | 2.62359E-05 | 0.044 |
|  | Lysinimonas_A_sp011620705 | 0 | 4.81357E-05 | 0.044 |
|  | Lysobacter_segetis | 0 | 4.01035E-05 | 0.044 |
|  | Lysobacter_sp004361065 | 0 | 1.88172E-05 | 0.044 |
|  | M3007_sp903905445 | 0 | 0.006865003 | 0.044 |
|  | MB11C04_sp002722545 | 0 | 0.003900777 | 0.044 |
|  | Mageeibacillus_indolicus | 0 | 3.61989E-06 | 0.044 |
|  | Malassezia_sympodialis | 0 | 2.34628E-05 | 0.044 |
|  | Marisediminicola_antarctica | 0 | 2.4184E-05 | 0.044 |
|  | Marisediminicola_sp014378715 | 0 | 4.38667E-06 | 0.044 |
|  | Massilia_oculi | 0 | 6.51401E-05 | 0.044 |
|  | Meiothermus_B_silvanus | 0 | 2.09712E-05 | 0.044 |
|  | Mesorhizobium_defluvii | 0 | 0.000151922 | 0.044 |
|  | Mesorhizobium_sp004791165 | 0 | 1.78713E-05 | 0.044 |
|  | Methylobacterium_sp001423085 | 0 | 5.25849E-05 | 0.044 |
|  | Methylophilus_medardicus | 0 | 0.012419722 | 0.044 |
|  | Methylophilus_methylotrophus | 0 | 0.091084935 | 0.044 |
|  | Methylophilus_sp001183865 | 0 | 0.074888636 | 0.044 |
|  | Methylophilus_sp001424665 | 0 | 0.005754297 | 0.044 |
|  | Methylophilus_sp008015755 | 0 | 0.128101894 | 0.044 |
|  | Methylotenera_A_sp002083635 | 0 | 0.00159227 | 0.044 |
|  | Methyloversatilis_universalis | 0 | 0.009972929 | 0.044 |
|  | Methyloversatilis_universalis_A | 0 | 1.17533E-05 | 0.044 |
|  | Microbacterium_A_agarici | 0 | 0.000232689 | 0.044 |
|  | Microbacterium_enclense | 0 | 4.11168E-05 | 0.044 |
|  | Microbacterium_enclense_A | 0 | 2.82888E-05 | 0.044 |
|  | Microbacterium_esteraromaticum_C | 0 | 7.7266E-05 | 0.044 |
|  | Microbacterium_gubbeenense | 0 | 1.77064E-05 | 0.044 |
|  | Microbacterium_hominis_B | 0 | 7.64084E-05 | 0.044 |
|  | Microbacterium_immunditiarum | 0 | 2.43921E-05 | 0.044 |
|  | Microbacterium_indicum | 0 | 6.91377E-05 | 0.044 |
|  | Microbacterium_invictum | 0 | 1.54802E-05 | 0.044 |
|  | Microbacterium_lacus | 0 | 0.000186392 | 0.044 |
|  | Microbacterium_mangrovi | 0 | 2.39739E-05 | 0.044 |
|  | Microbacterium_oleivorans | 0 | 1.72385E-05 | 0.044 |
|  | Microbacterium_oryzae | 0 | 0.000242931 | 0.044 |
|  | Microbacterium_phyllosphaerae | 0 | 3.23873E-05 | 0.044 |
|  | Microbacterium_protaetiae | 0 | 4.80075E-05 | 0.044 |
|  | Microbacterium_saccharophilum | 0 | 0.000323553 | 0.044 |
|  | Microbacterium_sp000411455 | 0 | 0.000111037 | 0.044 |
|  | Microbacterium_sp001049495 | 0 | 0.000105479 | 0.044 |
|  | Microbacterium_sp001314225 | 0 | 2.01768E-05 | 0.044 |
|  | Microbacterium_sp001423485 | 0 | 5.45667E-05 | 0.044 |
|  | Microbacterium_sp001427145 | 0 | 0.00010529 | 0.044 |
|  | Microbacterium_sp001428485 | 0 | 0.000885443 | 0.044 |
|  | Microbacterium_sp001639925 | 0 | 0.000210642 | 0.044 |
|  | Microbacterium_sp001897945 | 0 | 9.77547E-05 | 0.044 |
|  | Microbacterium_sp001898325 | 0 | 0.0001053 | 0.044 |
|  | Microbacterium_sp002245215 | 0 | 1.63327E-05 | 0.044 |
|  | Microbacterium_sp003075395 | 0 | 5.01341E-05 | 0.044 |
|  | Microbacterium_sp004366135 | 0 | 0.000104359 | 0.044 |
|  | Microbacterium_sp004794465 | 0 | 0.000206504 | 0.044 |
|  | Microbacterium_sp006715565 | 0 | 4.00526E-05 | 0.044 |
|  | Microbacterium_sp006715675 | 0 | 3.24563E-05 | 0.044 |
|  | Microbacterium_sp007667425 | 0 | 0.000531207 | 0.044 |
|  | Microbacterium_sp007828185 | 0 | 4.37115E-05 | 0.044 |
|  | Microbacterium_sp009649635 | 0 | 0.00012361 | 0.044 |
|  | Microbacterium_sp011326725 | 0 | 0.000114387 | 0.044 |
|  | Microbacterium_sp013409015 | 0 | 0.000202699 | 0.044 |
|  | Microbacterium_sp900156455 | 0 | 3.63894E-05 | 0.044 |
|  | Microbacterium_testaceum_F | 0 | 5.13617E-05 | 0.044 |
|  | Microbacterium_yannicii | 0 | 4.75259E-05 | 0.044 |
|  | Microcella_alkaliphila_A | 0 | 3.70834E-05 | 0.044 |
|  | Microterricola_sp000799285 | 0 | 2.52825E-05 | 0.044 |
|  | Mitsuokella_sp000469545 | 0 | 8.57338E-06 | 0.044 |
|  | Modestobacter_marinus_A | 0 | 5.8269E-05 | 0.044 |
|  | Mumia_xiangluensis | 0 | 0.001363318 | 0.044 |
|  | Mycobacterium_obuense | 0 | 0.004720723 | 0.044 |
|  | Neorhizobium_sp002500765 | 0 | 0.000129159 | 0.044 |
|  | Nocardia_nova_B | 0 | 0.000167164 | 0.044 |
|  | Novosphingobium_guangzhouense | 0 | 0.003210871 | 0.044 |
|  | Ochrobactrum_sp900470195 | 0 | 6.86715E-05 | 0.044 |
|  | Paenochrobactrum_gallinarii | 0 | 0.000133509 | 0.044 |
|  | Paraburkholderia_bannensis | 0 | 0.003089552 | 0.044 |
|  | Paraburkholderia_phenazinium_A | 0 | 8.2954E-06 | 0.044 |
|  | Paraburkholderia_sp900996235 | 0 | 0.004214683 | 0.044 |
|  | Paracoccus_homiensis | 0 | 6.20378E-05 | 0.044 |
|  | Paracoccus_ravus | 0 | 6.1652E-05 | 0.044 |
|  | Paracoccus_siganidrum | 0 | 0.00013159 | 0.044 |
|  | Paracoccus_sp002359815 | 0 | 5.53363E-05 | 0.044 |
|  | Paracoccus_thiocyanatus | 0 | 0.000129755 | 0.044 |
|  | Pararhizobium_sp003217095 | 0 | 2.67411E-05 | 0.044 |
|  | Pararhizobium_sp900067135 | 0 | 3.34936E-05 | 0.044 |
|  | Pauljensenia_sp900541895 | 0 | 0.000293789 | 0.044 |
|  | Pauljensenia_turicensis | 0 | 6.01208E-05 | 0.044 |
|  | Peptococcus_niger | 0 | 0.024143378 | 0.044 |
|  | Peptoniphilus_A_grossensis | 0 | 0.001964935 | 0.044 |
|  | Peptoniphilus_B_sp000478985 | 0 | 0.002193989 | 0.044 |
|  | Plantibacter_sp001423185 | 0 | 2.1252E-05 | 0.044 |
|  | Porphyromonas_A_somerae | 0 | 0.015016218 | 0.044 |
|  | Porphyromonas_asaccharolytica | 0 | 0.001483573 | 0.044 |
|  | Porphyromonas_sp900539155 | 0 | 0.000292532 | 0.044 |
|  | Porphyromonas_sp900546675 | 0 | 0.000212578 | 0.044 |
|  | Porphyromonas_sp900548415 | 0 | 0.004298855 | 0.044 |
|  | Prevotella_seregens | 0 | 0.012583208 | 0.044 |
|  | Prevotella_sp013333285 | 0 | 0.015593855 | 0.044 |
|  | Priestia_flexa | 0 | 0.000167737 | 0.044 |
|  | Propionimicrobium_sp900155645 | 0 | 7.30142E-05 | 0.044 |
|  | Protaetiibacter_sp014483895 | 0 | 4.06987E-05 | 0.044 |
|  | Pseudaminobacter_arsenicus | 0 | 4.31074E-05 | 0.044 |
|  | Pseudomonas_A_stutzeri_P | 0 | 1.45357E-05 | 0.044 |
|  | Pseudomonas_A_xanthomarina | 0 | 1.0996E-06 | 0.044 |
|  | Pseudomonas_E_abietaniphila | 0 | 5.49541E-05 | 0.044 |
|  | Pseudomonas_E_bohemica | 0 | 0.001576155 | 0.044 |
|  | Pseudomonas_E_chlororaphis_F | 0 | 0.001681781 | 0.044 |
|  | Pseudomonas_E_massiliensis | 0 | 3.84271E-05 | 0.044 |
|  | Pseudomonas_E_mendocina | 0 | 0.023330174 | 0.044 |
|  | Pseudoxanthomonas_A_kalamensis | 0 | 5.2968E-05 | 0.044 |
|  | Psychrobacter_sp002414005 | 0 | 0.000372471 | 0.044 |
|  | Puccinia_triticina | 0 | 0.003509436 | 0.044 |
|  | Pyramidobacter_piscolens | 0 | 8.93723E-06 | 0.044 |
|  | QEVD01_sp003576975 | 0 | 2.14421E-05 | 0.044 |
|  | Rhizorhabdus_sp004297635 | 0 | 0.009405834 | 0.044 |
|  | Rhodovarius_lipocyclicus | 0 | 0.010237153 | 0.044 |
|  | Rubrivivax_sp001725505 | 0 | 0.000400931 | 0.044 |
|  | SCN-69-89_sp008039575 | 0 | 1.25471E-05 | 0.044 |
|  | Saccharimonas_sp013333675 | 0 | 8.15722E-05 | 0.044 |
|  | Scardovia_wiggsiae | 0 | 0.006219987 | 0.044 |
|  | Shewanella_morhuae | 0 | 3.62951E-05 | 0.044 |
|  | Shinella_sp001713395 | 0 | 2.87824E-05 | 0.044 |
|  | Sphingobacterium_sp000938735 | 0 | 4.63404E-05 | 0.044 |
|  | Sphingobacterium_sp002734245 | 0 | 0.002015203 | 0.044 |
|  | Sphingobium_barthaii_A | 0 | 4.80721E-05 | 0.044 |
|  | Sphingomicrobium_rhizophila | 0 | 0.003599011 | 0.044 |
|  | Sphingomicrobium_sp902806285 | 0 | 7.33831E-05 | 0.044 |
|  | Sphingomonas_sp000251145 | 0 | 2.81273E-05 | 0.044 |
|  | Sphingomonas_sp002292295 | 0 | 8.73796E-06 | 0.044 |
|  | Sphingomonas_sp903884945 | 0 | 0.002149398 | 0.044 |
|  | Sphingopyxis_sp001468285 | 0 | 2.98454E-05 | 0.044 |
|  | Sphingopyxis_sp012035435 | 0 | 4.71542E-05 | 0.044 |
|  | Sphingosinicella_sp013911755 | 0 | 0.001570245 | 0.044 |
|  | Staphylococcus_saprophyticus | 0 | 0.000114384 | 0.044 |
|  | Stenotrophomonas_maltophilia_AN | 0 | 7.42499E-05 | 0.044 |
|  | Streptomyces_sp001984575 | 0 | 5.50017E-06 | 0.044 |
|  | Streptomyces_sp004193175 | 0 | 2.80193E-05 | 0.044 |
|  | Sutterella_sp900762445 | 0 | 0.005970276 | 0.044 |
|  | Tagaea_sp014444615 | 0 | 0.000972614 | 0.044 |
|  | Tatumella_citrea | 0 | 0.002901524 | 0.044 |
|  | Tersicoccus_phoenicis | 0 | 0.010285849 | 0.044 |
|  | Tersicoccus_sp001968825 | 0 | 6.5821E-05 | 0.044 |
|  | Thauera_propionica | 0 | 0.00094978 | 0.044 |
|  | Thermicanus_aegyptius | 0 | 0.002980375 | 0.044 |
|  | Thermomonas_fusca | 0 | 4.56174E-05 | 0.044 |
|  | Treponema_D_sp014334325 | 0 | 0.001644387 | 0.044 |
|  | Trichoderma_asperellum | 0 | 4.31257E-06 | 0.044 |
|  | UBA2030_sp002332755 | 0 | 0.02761059 | 0.044 |
|  | Varibaculum_massiliense | 0 | 0.063187115 | 0.044 |
|  | Veillonella_sp900757715 | 0 | 0.015989713 | 0.044 |
|  | Vibrio_fluvialis | 0 | 0.011668638 | 0.044 |
|  | Yokenella_regensburgei | 0 | 3.0193E-05 | 0.044 |
|  | ZYF759_sp012911015 | 0 | 3.69672E-05 | 0.044 |
|  | Zhihengliuella_salsuginis | 0 | 6.79553E-05 | 0.044 |
|  | Paracoccus_marinus | 0.111002039 | 0.019005939 | 0.045 |
|  | Pauljensenia_odontolytica | 0.091674353 | 0.069328048 | 0.046 |
|  | Ralstonia_sp000620465 | 15.15887812 | 11.12343742 | 0.046 |
|  | Janibacter_melonis | 0.052011176 | 0.011852428 | 0.048 |
|  | Gordonia_sputi | 0.062712875 | 0.124955315 | 0.049 |
|  | Caballeronia_udeis | 0.006928366 | 9.78639E-07 | 0.050 |
|  | Sphingomonas_paucimobilis | 0.494687028 | 0.193116139 | 0.000 |
|  | Pelomonas_puraquae | 1.487479778 | 0.238642014 | 0.000 |
|  | Escherichia_sp001660175 | 0 | 8.37169E-05 | 0.000 |
|  | Acinetobacter_guillouiae | 0.537353134 | 0.053845222 | 0.001 |
|  | Lawsonella_clevelandensis_A | 1.343853118 | 0.478341111 | 0.001 |
|  | Pelomonas_sp003963075 | 0.062634937 | 3.96689E-05 | 0.001 |
|  | Acinetobacter_johnsonii | 1.162154686 | 0.470502757 | 0.003 |
|  | Paraburkholderia_ferrariae | 0.034797257 | 0.010409431 | 0.003 |
|  | Escherichia_fergusonii | 0 | 0.000163834 | 0.004 |
|  | Microbacterium_sp000383475 | 0 | 0.004926742 | 0.004 |
|  | Peptoniphilus_C_coxii | 0 | 0.031061134 | 0.004 |
|  | Rhodococcus_ruber | 0 | 0.005054519 | 0.004 |
|  | Staphylococcus_A_sciuri | 0 | 0.011786662 | 0.004 |
|  | Geobacillus_thermoleovorans | 3.437051358 | 0.764884275 | 0.005 |
|  | Cutibacterium_granulosum | 0.46349832 | 0.157418854 | 0.007 |
|  | Aquabacterium_parvum | 0.043070006 | 0.005018397 | 0.008 |
|  | Pseudonocardia_ammonioxydans | 0.227270472 | 0.112806409 | 0.010 |
|  | Ralstonia_pickettii | 5.894606646 | 3.494152491 | 0.010 |
|  | Corynebacterium_accolens | 0.354276247 | 0.106820578 | 0.010 |
|  | Burkholderia_ubonensis | 0.27184265 | 0.128353722 | 0.011 |
|  | Microbacterium_lacticum | 0.12971828 | 0.018376148 | 0.012 |
|  | Ralstonia_sp000801955 | 0.221604889 | 0.099933127 | 0.013 |
|  | Pseudomonas_E_carnis | 0.561318547 | 0.260162385 | 0.017 |
|  | Janibacter_anophelis | 0.152010097 | 0.047526547 | 0.017 |
|  | Perlucidibaca_sp002943415 | 0.95470969 | 0.52928345 | 0.018 |
|  | Escherichia_albertii | 3.66924E-05 | 0.000257681 | 0.020 |
|  | Kocuria_palustris | 0.296023128 | 0.086735002 | 0.021 |
|  | Cellvibrio_sp008806975 | 0.010979842 | 0 | 0.021 |
|  | Comamonas_koreensis | 0.000211768 | 0.001175156 | 0.023 |
|  | Escherichia_sp005843885 | 5.61211E-05 | 0.000226528 | 0.023 |
|  | Ralstonia_insidiosa | 0.330265197 | 0.202872635 | 0.024 |
|  | Brevibacillus_D_fluminis | 0.013715663 | 0 | 0.026 |
|  | Burkholderia_vietnamiensis | 0.198346109 | 0.075410303 | 0.026 |
|  | Sphingobium_yanoikuyae | 0.111284731 | 0.059849681 | 0.026 |
|  | Pseudomonas_aeruginosa | 0.63106464 | 0.39070812 | 0.028 |
|  | Gordonia_jacobaea | 0.029765654 | 0.01412621 | 0.029 |
|  | Rhodococcus_qingshengii | 0.277853964 | 0.122046166 | 0.034 |
|  | Serinicoccus_profundi | 0.229609002 | 0.029940812 | 0.035 |
|  | Bordetella_B_ansorpii_B | 0.167339602 | 0.104092816 | 0.037 |
|  | Leucobacter_sp900163635 | 2.40495E-05 | 0.00091041 | 0.037 |
|  | Stenotrophomonas_maltophilia | 5.86535E-05 | 0.002334596 | 0.037 |
|  | Burkholderia_oklahomensis | 0.071746515 | 0.023312567 | 0.037 |
|  | Saccharomonospora_isguenensis | 0.005882786 | 0 | 0.038 |
|  | Bosea_sp900156025 | 9.21406E-05 | 0.000639676 | 0.039 |
|  | Microbacterium_sp011046485 | 3.69564E-05 | 0.004407246 | 0.039 |
|  | Capnocytophaga_granulosa | 0.002871533 | 0.005776051 | 0.042 |
|  | Paracoccus_sp009674885 | 4.95296E-05 | 0.0001144 | 0.042 |
|  | Prevotella_sp000467895 | 0.002733698 | 0.005018658 | 0.042 |
|  | Stenotrophomonas_maltophilia_AL | 0.015993446 | 0.000192527 | 0.042 |
|  | Burkholderia_sp000687455 | 0.105740556 | 0.037049996 | 0.043 |
|  | 13-2-20CM-66-19_sp003136935 | 0 | 8.9258E-07 | 0.044 |
|  | ALPHA2B_sp005503065 | 0 | 2.12109E-05 | 0.044 |
|  | Acidovorax_soli_A | 0 | 0.051100919 | 0.044 |
|  | Acidovorax_sp003208485 | 0 | 0.008855658 | 0.044 |
|  | Acidovorax_sp003852545 | 0 | 0.001714897 | 0.044 |
|  | Acidovorax_sp005405905 | 0 | 0.005245225 | 0.044 |
|  | Acidovorax_sp013408765 | 0 | 0.036192634 | 0.044 |
|  | Actinobaculum_massiliense | 0 | 0.078184853 | 0.044 |
|  | Actinotignum_sanguinis | 0 | 0.004095909 | 0.044 |
|  | Actinotignum_schaalii | 0 | 0.016494105 | 0.044 |
|  | Aerococcus_urinae_C | 0 | 0.011195571 | 0.044 |
|  | Aerococcus_urinae_D | 0 | 0.381710681 | 0.044 |
|  | Aeromicrobium_sp000471045 | 0 | 7.51801E-05 | 0.044 |
|  | Aeromicrobium_sp002174305 | 0 | 2.71589E-05 | 0.044 |
|  | Aeromonas_sanarellii | 0 | 0.002577966 | 0.044 |
|  | Agrococcus_sp005484985 | 0 | 6.96725E-05 | 0.044 |
|  | Agromyces_italicus | 0 | 2.42256E-05 | 0.044 |
|  | Alcaligenes_phenolicus | 0 | 0.000111018 | 0.044 |
|  | Alloscardovia_omnicolens | 0 | 0.000149558 | 0.044 |
|  | Amaricoccus_macauensis | 0 | 5.80438E-05 | 0.044 |
|  | Aminobacter_niigataensis | 0 | 6.56717E-05 | 0.044 |
|  | Anaerococcus_vaginalis_B | 0 | 0.029922566 | 0.044 |
|  | Antricoccus_suffuscus | 0 | 2.64911E-05 | 0.044 |
|  | Aquabacterium_commune | 0 | 0.023699235 | 0.044 |
|  | Aquabacterium_sp004310865 | 0 | 3.57361E-05 | 0.044 |
|  | Aquabacterium_sp903894125 | 0 | 0.004599896 | 0.044 |
|  | Aspergillus_terreus | 0 | 0.001309954 | 0.044 |
|  | Bacillus_J_thermoamylovorans | 0 | 0.028272664 | 0.044 |
|  | Bacteroides_sp900552405 | 0 | 0.000516317 | 0.044 |
|  | Bacteroides_stercorirosoris | 0 | 0.000101009 | 0.044 |
|  | Bacteroides_uniformis | 0 | 0.017061005 | 0.044 |
|  | Bifidobacterium_sp003585735 | 0 | 2.06468E-05 | 0.044 |
|  | Bifidobacterium_vaginale_D | 0 | 0.002636299 | 0.044 |
|  | Bifidobacterium_vaginale_G | 0 | 0.000230612 | 0.044 |
|  | Blastococcus_sp003319185 | 0 | 1.879E-05 | 0.044 |
|  | Blautia_A_massiliensis | 0 | 0.007004622 | 0.044 |
|  | Bordetella_C_sp002261215 | 0 | 6.00369E-05 | 0.044 |
|  | Bosea_sp005502805 | 0 | 0.00112705 | 0.044 |
|  | Bosea_sp008253865 | 0 | 0.000536116 | 0.044 |
|  | Bosea_sp011764485 | 0 | 8.62578E-05 | 0.044 |
|  | Bowdeniella_nasicola | 0 | 2.64749E-05 | 0.044 |
|  | Bowdeniella_nasicola_A | 0 | 6.44182E-05 | 0.044 |
|  | Brachybacterium_faecium | 0 | 0.000107707 | 0.044 |
|  | Brachybacterium_saurashtrense | 0 | 0.000111612 | 0.044 |
|  | Bradyrhizobium_guangzhouense | 0 | 0.000714123 | 0.044 |
|  | Brevundimonas_sp002157625 | 0 | 4.17722E-05 | 0.044 |
|  | Brucella_melitensis | 0 | 4.3428E-05 | 0.044 |
|  | Burkholderia_lata | 0 | 4.75043E-07 | 0.044 |
|  | Burkholderia_sp003635165 | 0 | 0.008361092 | 0.044 |
|  | Burkholderia_sp902833225 | 0 | 1.43562E-06 | 0.044 |
|  | CADCTG01_sp902805645 | 0 | 0.00198098 | 0.044 |
|  | CAG-873_sp009775535 | 0 | 0.000100485 | 0.044 |
|  | Caballeronia_insecticola | 0 | 2.24584E-05 | 0.044 |
|  | Campylobacter_B_ureolyticus | 0 | 0.025892124 | 0.044 |
|  | Campylobacter_B_ureolyticus_A | 0 | 0.00669076 | 0.044 |
|  | Caulobacter_sp000426025 | 0 | 4.82609E-05 | 0.044 |
|  | Caulobacter_sp004144935 | 0 | 4.08137E-05 | 0.044 |
|  | Centipeda_sp000468035 | 0 | 0.000107107 | 0.044 |
|  | Cereibacter_A_sphaeroides | 0 | 8.42726E-05 | 0.044 |
|  | Chlamydophila_avium | 0 | 0.000206056 | 0.044 |
|  | Citrobacter_freundii | 0 | 7.13856E-05 | 0.044 |
|  | Citrobacter_murliniae | 0 | 1.09432E-05 | 0.044 |
|  | Citrobacter_portucalensis_A | 0 | 1.78982E-05 | 0.044 |
|  | Cohnella_phaseoli | 0 | 1.87409E-05 | 0.044 |
|  | Comamonas_composti | 0 | 4.87735E-05 | 0.044 |
|  | Comamonas_piscis | 0 | 3.27064E-05 | 0.044 |
|  | Comamonas_testosteroni_B | 0 | 3.63801E-05 | 0.044 |
|  | Conyzicola_nivalis | 0 | 3.82405E-05 | 0.044 |
|  | Corynebacterium_frankenforstense | 0 | 0.003531117 | 0.044 |
|  | Corynebacterium_pollutisoli | 0 | 8.77107E-05 | 0.044 |
|  | Corynebacterium_sp001807265 | 0 | 0.002827541 | 0.044 |
|  | Corynebacterium_sp001875665 | 0 | 0.000407508 | 0.044 |
|  | Corynebacterium_sp001875725 | 0 | 0.002700703 | 0.044 |
|  | Corynebacterium_sp014490595 | 0 | 4.51211E-05 | 0.044 |
|  | Cumulibacter_manganitolerans | 0 | 9.54746E-05 | 0.044 |
|  | Curtobacterium_luteum_A | 0 | 0.002316184 | 0.044 |
|  | Curtobacterium_sp001864895 | 0 | 0.00070559 | 0.044 |
|  | DSM-21351_sp002250625 | 0 | 1.35662E-06 | 0.044 |
|  | Devosia_sp001425445 | 0 | 1.61377E-05 | 0.044 |
|  | Dialister_B_micraerophilus | 0 | 0.00234483 | 0.044 |
|  | Dyadobacter_sp001898145 | 0 | 0.004722873 | 0.044 |
|  | Eikenella_corrodens | 0 | 0.001517239 | 0.044 |
|  | Ensifer_sp900469595 | 0 | 1.93346E-05 | 0.044 |
|  | Enterococcus_E_cecorum | 0 | 0.038518462 | 0.044 |
|  | Epilithonimonas_sp003932955 | 0 | 7.56175E-05 | 0.044 |
|  | Erwinia_aphidicola | 0 | 5.76314E-06 | 0.044 |
|  | Erythrobacter_cryptus | 0 | 0.002117358 | 0.044 |
|  | Fastidiosipila_sanguinis | 0 | 0.006210832 | 0.044 |
|  | Franconibacter_helveticus | 0 | 4.6348E-05 | 0.044 |
|  | Frigoribacterium_faeni_A | 0 | 0.00683074 | 0.044 |
|  | Gandjariella_thermophila | 0 | 1.01887E-05 | 0.044 |
|  | Gemmobacter_B_sp002855575 | 0 | 5.01371E-05 | 0.044 |
|  | Granulicatella_sp001058355 | 0 | 8.92235E-05 | 0.044 |
|  | Gryllotalpicola_sp009780695 | 0 | 7.10111E-05 | 0.044 |
|  | Halolamina_pelagica | 0 | 3.46288E-05 | 0.044 |
|  | Herbiconiux_sp004297105 | 0 | 0.000142299 | 0.044 |
|  | Im94_sp009749525 | 0 | 2.94824E-05 | 0.044 |
|  | Kaistia_adipata | 0 | 1.25554E-05 | 0.044 |
|  | Klebsiella_A_michiganensis_B | 0 | 0.005132596 | 0.044 |
|  | Klebsiella_quasivariicola | 0 | 0.00046118 | 0.044 |
|  | Knoellia_remsis | 0 | 2.46063E-05 | 0.044 |
|  | Kocuria_atrinae | 0 | 0.000342606 | 0.044 |
|  | Lacisediminihabitans_profunda | 0 | 2.7441E-05 | 0.044 |
|  | Lactobacillus_crispatus | 0 | 0.31181548 | 0.044 |
|  | Lactobacillus_johnsonii | 0 | 0.000229763 | 0.044 |
|  | Lawsonella_clevelandensis | 0 | 0.000147249 | 0.044 |
|  | Leifsonia_aquatica_A | 0 | 4.6689E-05 | 0.044 |
|  | Leifsonia_sp002105485 | 0 | 2.50812E-05 | 0.044 |
|  | Leucobacter_sp000980875 | 0 | 0.000101622 | 0.044 |
|  | Leucobacter_sp002752355 | 0 | 3.88405E-05 | 0.044 |
|  | Leucobacter_sp014529985 | 0 | 2.92155E-05 | 0.044 |
|  | Leuconostoc_inhae | 0 | 0.000373234 | 0.044 |
|  | Luteimonas_sp002307375 | 0 | 2.62359E-05 | 0.044 |
|  | Lysinimonas_A_sp011620705 | 0 | 4.81357E-05 | 0.044 |
|  | Lysobacter_segetis | 0 | 4.01035E-05 | 0.044 |
|  | Lysobacter_sp004361065 | 0 | 1.88172E-05 | 0.044 |
|  | M3007_sp903905445 | 0 | 0.006865003 | 0.044 |
|  | MB11C04_sp002722545 | 0 | 0.003900777 | 0.044 |
|  | Mageeibacillus_indolicus | 0 | 3.61989E-06 | 0.044 |
|  | Malassezia_sympodialis | 0 | 2.34628E-05 | 0.044 |
|  | Marisediminicola_antarctica | 0 | 2.4184E-05 | 0.044 |
|  | Marisediminicola_sp014378715 | 0 | 4.38667E-06 | 0.044 |
|  | Massilia_oculi | 0 | 6.51401E-05 | 0.044 |
|  | Meiothermus_B_silvanus | 0 | 2.09712E-05 | 0.044 |
|  | Mesorhizobium_defluvii | 0 | 0.000151922 | 0.044 |
|  | Mesorhizobium_sp004791165 | 0 | 1.78713E-05 | 0.044 |
|  | Methylobacterium_sp001423085 | 0 | 5.25849E-05 | 0.044 |
|  | Methylophilus_medardicus | 0 | 0.012419722 | 0.044 |
|  | Methylophilus_methylotrophus | 0 | 0.091084935 | 0.044 |
|  | Methylophilus_sp001183865 | 0 | 0.074888636 | 0.044 |
|  | Methylophilus_sp001424665 | 0 | 0.005754297 | 0.044 |
|  | Methylophilus_sp008015755 | 0 | 0.128101894 | 0.044 |
|  | Methylotenera_A_sp002083635 | 0 | 0.00159227 | 0.044 |
|  | Methyloversatilis_universalis | 0 | 0.009972929 | 0.044 |
|  | Methyloversatilis_universalis_A | 0 | 1.17533E-05 | 0.044 |
|  | Microbacterium_A_agarici | 0 | 0.000232689 | 0.044 |
|  | Microbacterium_enclense | 0 | 4.11168E-05 | 0.044 |
|  | Microbacterium_enclense_A | 0 | 2.82888E-05 | 0.044 |
|  | Microbacterium_esteraromaticum_C | 0 | 7.7266E-05 | 0.044 |
|  | Microbacterium_gubbeenense | 0 | 1.77064E-05 | 0.044 |
|  | Microbacterium_hominis_B | 0 | 7.64084E-05 | 0.044 |
|  | Microbacterium_immunditiarum | 0 | 2.43921E-05 | 0.044 |
|  | Microbacterium_indicum | 0 | 6.91377E-05 | 0.044 |
|  | Microbacterium_invictum | 0 | 1.54802E-05 | 0.044 |
|  | Microbacterium_lacus | 0 | 0.000186392 | 0.044 |
|  | Microbacterium_mangrovi | 0 | 2.39739E-05 | 0.044 |
|  | Microbacterium_oleivorans | 0 | 1.72385E-05 | 0.044 |
|  | Microbacterium_oryzae | 0 | 0.000242931 | 0.044 |
|  | Microbacterium_phyllosphaerae | 0 | 3.23873E-05 | 0.044 |
|  | Microbacterium_protaetiae | 0 | 4.80075E-05 | 0.044 |
|  | Microbacterium_saccharophilum | 0 | 0.000323553 | 0.044 |
|  | Microbacterium_sp000411455 | 0 | 0.000111037 | 0.044 |
|  | Microbacterium_sp001049495 | 0 | 0.000105479 | 0.044 |
|  | Microbacterium_sp001314225 | 0 | 2.01768E-05 | 0.044 |
|  | Microbacterium_sp001423485 | 0 | 5.45667E-05 | 0.044 |
|  | Microbacterium_sp001427145 | 0 | 0.00010529 | 0.044 |
|  | Microbacterium_sp001428485 | 0 | 0.000885443 | 0.044 |
|  | Microbacterium_sp001639925 | 0 | 0.000210642 | 0.044 |
|  | Microbacterium_sp001897945 | 0 | 9.77547E-05 | 0.044 |
|  | Microbacterium_sp001898325 | 0 | 0.0001053 | 0.044 |
|  | Microbacterium_sp002245215 | 0 | 1.63327E-05 | 0.044 |
|  | Microbacterium_sp003075395 | 0 | 5.01341E-05 | 0.044 |
|  | Microbacterium_sp004366135 | 0 | 0.000104359 | 0.044 |
|  | Microbacterium_sp004794465 | 0 | 0.000206504 | 0.044 |
|  | Microbacterium_sp006715565 | 0 | 4.00526E-05 | 0.044 |
|  | Microbacterium_sp006715675 | 0 | 3.24563E-05 | 0.044 |
|  | Microbacterium_sp007667425 | 0 | 0.000531207 | 0.044 |
|  | Microbacterium_sp007828185 | 0 | 4.37115E-05 | 0.044 |
|  | Microbacterium_sp009649635 | 0 | 0.00012361 | 0.044 |
|  | Microbacterium_sp011326725 | 0 | 0.000114387 | 0.044 |
|  | Microbacterium_sp013409015 | 0 | 0.000202699 | 0.044 |
|  | Microbacterium_sp900156455 | 0 | 3.63894E-05 | 0.044 |
|  | Microbacterium_testaceum_F | 0 | 5.13617E-05 | 0.044 |
|  | Microbacterium_yannicii | 0 | 4.75259E-05 | 0.044 |
|  | Microcella_alkaliphila_A | 0 | 3.70834E-05 | 0.044 |
|  | Microterricola_sp000799285 | 0 | 2.52825E-05 | 0.044 |
|  | Mitsuokella_sp000469545 | 0 | 8.57338E-06 | 0.044 |
|  | Modestobacter_marinus_A | 0 | 5.8269E-05 | 0.044 |
|  | Mumia_xiangluensis | 0 | 0.001363318 | 0.044 |
|  | Mycobacterium_obuense | 0 | 0.004720723 | 0.044 |
|  | Neorhizobium_sp002500765 | 0 | 0.000129159 | 0.044 |
|  | Nocardia_nova_B | 0 | 0.000167164 | 0.044 |
|  | Novosphingobium_guangzhouense | 0 | 0.003210871 | 0.044 |
|  | Ochrobactrum_sp900470195 | 0 | 6.86715E-05 | 0.044 |
|  | Paenochrobactrum_gallinarii | 0 | 0.000133509 | 0.044 |
|  | Paraburkholderia_bannensis | 0 | 0.003089552 | 0.044 |
|  | Paraburkholderia_phenazinium_A | 0 | 8.2954E-06 | 0.044 |
|  | Paraburkholderia_sp900996235 | 0 | 0.004214683 | 0.044 |
|  | Paracoccus_homiensis | 0 | 6.20378E-05 | 0.044 |
|  | Paracoccus_ravus | 0 | 6.1652E-05 | 0.044 |
|  | Paracoccus_siganidrum | 0 | 0.00013159 | 0.044 |
|  | Paracoccus_sp002359815 | 0 | 5.53363E-05 | 0.044 |
|  | Paracoccus_thiocyanatus | 0 | 0.000129755 | 0.044 |
|  | Pararhizobium_sp003217095 | 0 | 2.67411E-05 | 0.044 |
|  | Pararhizobium_sp900067135 | 0 | 3.34936E-05 | 0.044 |
|  | Pauljensenia_sp900541895 | 0 | 0.000293789 | 0.044 |
|  | Pauljensenia_turicensis | 0 | 6.01208E-05 | 0.044 |
|  | Peptococcus_niger | 0 | 0.024143378 | 0.044 |
|  | Peptoniphilus_A_grossensis | 0 | 0.001964935 | 0.044 |
|  | Peptoniphilus_B_sp000478985 | 0 | 0.002193989 | 0.044 |
|  | Plantibacter_sp001423185 | 0 | 2.1252E-05 | 0.044 |
|  | Porphyromonas_A_somerae | 0 | 0.015016218 | 0.044 |
|  | Porphyromonas_asaccharolytica | 0 | 0.001483573 | 0.044 |
|  | Porphyromonas_sp900539155 | 0 | 0.000292532 | 0.044 |
|  | Porphyromonas_sp900546675 | 0 | 0.000212578 | 0.044 |
|  | Porphyromonas_sp900548415 | 0 | 0.004298855 | 0.044 |
|  | Prevotella_seregens | 0 | 0.012583208 | 0.044 |
|  | Prevotella_sp013333285 | 0 | 0.015593855 | 0.044 |
|  | Priestia_flexa | 0 | 0.000167737 | 0.044 |
|  | Propionimicrobium_sp900155645 | 0 | 7.30142E-05 | 0.044 |
|  | Protaetiibacter_sp014483895 | 0 | 4.06987E-05 | 0.044 |
|  | Pseudaminobacter_arsenicus | 0 | 4.31074E-05 | 0.044 |
|  | Pseudomonas_A_stutzeri_P | 0 | 1.45357E-05 | 0.044 |
|  | Pseudomonas_A_xanthomarina | 0 | 1.0996E-06 | 0.044 |
|  | Pseudomonas_E_abietaniphila | 0 | 5.49541E-05 | 0.044 |
|  | Pseudomonas_E_bohemica | 0 | 0.001576155 | 0.044 |
|  | Pseudomonas_E_chlororaphis_F | 0 | 0.001681781 | 0.044 |
|  | Pseudomonas_E_massiliensis | 0 | 3.84271E-05 | 0.044 |
|  | Pseudomonas_E_mendocina | 0 | 0.023330174 | 0.044 |
|  | Pseudoxanthomonas_A_kalamensis | 0 | 5.2968E-05 | 0.044 |
|  | Psychrobacter_sp002414005 | 0 | 0.000372471 | 0.044 |
|  | Puccinia_triticina | 0 | 0.003509436 | 0.044 |
|  | Pyramidobacter_piscolens | 0 | 8.93723E-06 | 0.044 |
|  | QEVD01_sp003576975 | 0 | 2.14421E-05 | 0.044 |
|  | Rhizorhabdus_sp004297635 | 0 | 0.009405834 | 0.044 |
|  | Rhodovarius_lipocyclicus | 0 | 0.010237153 | 0.044 |
|  | Rubrivivax_sp001725505 | 0 | 0.000400931 | 0.044 |
|  | SCN-69-89_sp008039575 | 0 | 1.25471E-05 | 0.044 |
|  | Saccharimonas_sp013333675 | 0 | 8.15722E-05 | 0.044 |
|  | Scardovia_wiggsiae | 0 | 0.006219987 | 0.044 |
|  | Shewanella_morhuae | 0 | 3.62951E-05 | 0.044 |
|  | Shinella_sp001713395 | 0 | 2.87824E-05 | 0.044 |
|  | Sphingobacterium_sp000938735 | 0 | 4.63404E-05 | 0.044 |
|  | Sphingobacterium_sp002734245 | 0 | 0.002015203 | 0.044 |
|  | Sphingobium_barthaii_A | 0 | 4.80721E-05 | 0.044 |
|  | Sphingomicrobium_rhizophila | 0 | 0.003599011 | 0.044 |
|  | Sphingomicrobium_sp902806285 | 0 | 7.33831E-05 | 0.044 |
|  | Sphingomonas_sp000251145 | 0 | 2.81273E-05 | 0.044 |
|  | Sphingomonas_sp002292295 | 0 | 8.73796E-06 | 0.044 |
|  | Sphingomonas_sp903884945 | 0 | 0.002149398 | 0.044 |
|  | Sphingopyxis_sp001468285 | 0 | 2.98454E-05 | 0.044 |
|  | Sphingopyxis_sp012035435 | 0 | 4.71542E-05 | 0.044 |
|  | Sphingosinicella_sp013911755 | 0 | 0.001570245 | 0.044 |
|  | Staphylococcus_saprophyticus | 0 | 0.000114384 | 0.044 |
|  | Stenotrophomonas_maltophilia_AN | 0 | 7.42499E-05 | 0.044 |
|  | Streptomyces_sp001984575 | 0 | 5.50017E-06 | 0.044 |
|  | Streptomyces_sp004193175 | 0 | 2.80193E-05 | 0.044 |
|  | Sutterella_sp900762445 | 0 | 0.005970276 | 0.044 |
|  | Tagaea_sp014444615 | 0 | 0.000972614 | 0.044 |
|  | Tatumella_citrea | 0 | 0.002901524 | 0.044 |
|  | Tersicoccus_phoenicis | 0 | 0.010285849 | 0.044 |
|  | Tersicoccus_sp001968825 | 0 | 6.5821E-05 | 0.044 |
|  | Thauera_propionica | 0 | 0.00094978 | 0.044 |
|  | Thermicanus_aegyptius | 0 | 0.002980375 | 0.044 |
|  | Thermomonas_fusca | 0 | 4.56174E-05 | 0.044 |
|  | Treponema_D_sp014334325 | 0 | 0.001644387 | 0.044 |
|  | Trichoderma_asperellum | 0 | 4.31257E-06 | 0.044 |
|  | UBA2030_sp002332755 | 0 | 0.02761059 | 0.044 |
|  | Varibaculum_massiliense | 0 | 0.063187115 | 0.044 |
|  | Veillonella_sp900757715 | 0 | 0.015989713 | 0.044 |
|  | Vibrio_fluvialis | 0 | 0.011668638 | 0.044 |
|  | Yokenella_regensburgei | 0 | 3.0193E-05 | 0.044 |
|  | ZYF759_sp012911015 | 0 | 3.69672E-05 | 0.044 |
|  | Zhihengliuella_salsuginis | 0 | 6.79553E-05 | 0.044 |
|  | Paracoccus_marinus | 0.111002039 | 0.019005939 | 0.045 |
|  | Pauljensenia_odontolytica | 0.091674353 | 0.069328048 | 0.046 |
|  | Ralstonia_sp000620465 | 15.15887812 | 11.12343742 | 0.046 |
|  | Janibacter_melonis | 0.052011176 | 0.011852428 | 0.048 |
|  | Gordonia_sputi | 0.062712875 | 0.124955315 | 0.049 |
|  | Caballeronia_udeis | 0.006928366 | 9.78639E-07 | 0.050 |
