# Supplementary material for: Renal pelvis urobiome dysbiosis is associated with postoperative systemic inflammatory response syndrome after percutaneous nephrolithotomy
Source: mSystems. 2025 Aug 15;10(9):e00780-25. doi: 10.1128/msystems.00780-25 (PMC12455945; doi:10.1128/msystems.00780-25)
Supplement: Table S2 — Comparison of average abundance of predicted functions between SIRS(−) and SIRS(+). [file msystems.00780-25-s0002.docx]

Table S2. Comparison of average abundance of predicted functions between SIRS(-) and SIRS(+)

| KEGG | Average abundance | | |
| --- | --- | --- | --- |
|  | SIRS(-) | SIRS(+) | P value |
| Calcium signaling pathway | 13413.286 | 5696.947 | 0.000 |
| Retrograde endocannabinoid signaling | 19702.000 | 3774.421 | 0.000 |
| Isoflavonoid biosynthesis | 28523.416 | 9485.105 | 0.000 |
| Staurosporine biosynthesis | 257532.831 | 101396.526 | 0.001 |
| Systemic lupus erythematosus | 11506.104 | 4001.579 | 0.001 |
| Endocytosis | 9659.416 | 3227.316 | 0.001 |
| Type I polyketide structures | 19084.987 | 6514.211 | 0.001 |
| Flavonoid biosynthesis | 7018.870 | 2796.632 | 0.002 |
| Stilbenoid, diarylheptanoid and gingerol biosynthesis | 7018.870 | 2796.632 | 0.002 |
| Tetracycline biosynthesis | 18870.727 | 9565.211 | 0.002 |
| Platelet activation | 3141.792 | 1210.211 | 0.002 |
| Relaxin signaling pathway | 3048.532 | 1210.000 | 0.002 |
| Biosynthesis of type II polyketide products | 24923.558 | 12765.474 | 0.002 |
| ECM-receptor interaction | 5784.818 | 2388.105 | 0.003 |
| Sphingolipid signaling pathway | 39377.247 | 18670.000 | 0.003 |
| AGE-RAGE signaling pathway in diabetic complications | 2976.221 | 1210.000 | 0.003 |
| Cell adhesion molecules (CAMs) | 5707.987 | 2387.368 | 0.003 |
| Biosynthesis of type II polyketide backbone | 2511.844 | 157.158 | 0.004 |
| Caffeine metabolism | 15930.558 | 7123.158 | 0.004 |
| Alcoholism | 271506.766 | 154974.684 | 0.004 |
| Amphetamine addiction | 271523.675 | 154974.684 | 0.004 |
| Cocaine addiction | 271468.286 | 154974.684 | 0.004 |
| Dopaminergic synapse | 271978.519 | 155137.368 | 0.004 |
| Serotonergic synapse | 271578.558 | 155029.526 | 0.004 |
| Steroid degradation | 408344.247 | 208909.105 | 0.004 |
| Biosynthesis of enediyne antibiotics | 1556.286 | 1528.263 | 0.005 |
| Hematopoietic cell lineage | 13227.896 | 6291.684 | 0.006 |
| Neuroactive ligand-receptor interaction | 5110.753 | 1356.895 | 0.007 |
| Malaria | 7410.377 | 3310.895 | 0.008 |
| Bisphenol degradation | 14977.403 | 8212.684 | 0.009 |
| Morphine addiction | 4672.662 | 1320.000 | 0.009 |
| Bile secretion | 13598.494 | 16970.053 | 0.010 |
| Nicotine addiction | 4600.351 | 1320.000 | 0.010 |
| Renin-angiotensin system | 371680.610 | 193489.211 | 0.010 |
| Focal adhesion | 537.195 | 23.474 | 0.010 |
| Sesquiterpenoid and triterpenoid biosynthesis | 59965.584 | 37792.895 | 0.010 |
| Arrhythmogenic right ventricular cardiomyopathy (ARVC) | 503.558 | 22.947 | 0.011 |
| Regulation of actin cytoskeleton | 515.597 | 49.316 | 0.012 |
| Steroid biosynthesis | 41723.909 | 25606.316 | 0.012 |
| Insect hormone biosynthesis | 1725101.805 | 1114328.842 | 0.014 |
| GnRH signaling pathway | 2118.494 | 852.316 | 0.015 |
| Ras signaling pathway | 2118.494 | 852.316 | 0.015 |
| Pancreatic cancer | 2007.701 | 852.316 | 0.016 |
| Non-homologous end-joining | 1162376.481 | 752732.053 | 0.019 |
| Fc gamma R-mediated phagocytosis | 2613.675 | 1194.053 | 0.020 |
| Staphylococcus aureus infection | 807007.948 | 502234.368 | 0.023 |
| Tight junction | 3044.273 | 656.947 | 0.024 |
| Melanogenesis | 40651.351 | 11337.895 | 0.024 |
| Furfural degradation | 388738.831 | 260235.316 | 0.024 |
| Steroid hormone biosynthesis | 43510.468 | 28843.316 | 0.025 |
| Peroxisome | 8615339.571 | 7510491.526 | 0.029 |
| Mineral absorption | 2126380.636 | 1705686.000 | 0.030 |
| N-Glycan biosynthesis | 351653.169 | 246568.316 | 0.031 |
| cAMP signaling pathway | 120003.740 | 95927.263 | 0.035 |
| Nonribosomal peptide structures | 244451.481 | 178061.000 | 0.036 |
| Chloroalkane and chloroalkene degradation | 5952951.935 | 4686724.632 | 0.039 |
| Hypertrophic cardiomyopathy (HCM) | 56012.429 | 23188.632 | 0.039 |
| Indole alkaloid biosynthesis | 28016.468 | 14510.158 | 0.042 |
| Photosynthesis - antenna proteins | 43413.416 | 47074.316 | 0.044 |
| Oxytocin signaling pathway | 2396.844 | 368.842 | 0.045 |
| Jak-STAT signaling pathway | 0.000 | 0.368 | 0.047 |
| Renin secretion | 54913.078 | 22989.053 | 0.049 |
| Carotenoid biosynthesis | 804932.260 | 553832.632 | 0.049 |
| Prolactin signaling pathway | 262521.519 | 419159.421 | 0.049 |
